# SCALING: plug-n-play device-free indoor tracking

**DOI:** 10.1038/s41598-024-53524-z

**Published:** 2024-02-05

**Authors:** Zongxing Xie, Fan Ye

**Affiliations:** Electrical and Computer Engineering Department, Stony Brook University, Stony Brook, 11794 USA

**Keywords:** Electrical and electronic engineering, Occupational health

## Abstract

24/7 continuous recording of in-home daily trajectories is informative for health status assessment (e.g., monitoring Alzheimer’s, dementia based on behavior patterns). Indoor device-free localization/tracking are ideal because no user efforts on wearing devices are needed. However, prior work mainly focused on improving the localization accuracy. They relied on well-calibrated sensor placements, which require hours of intensive manual setup and respective expertise, feasible only at small scale and by mostly researchers themselves. Scaling the deployments to tens or hundreds of real homes, however, would incur prohibitive manual efforts, and become infeasible for layman users. We present *SCALING*, a plug-and-play indoor trajectory monitoring system that layman users can easily set up by walking a one-minute loop trajectory after placing radar nodes on walls. It uses a self calibrating algorithm that estimates sensor locations through their distance measurements to the person walking the trajectory, a trivial effort without taxing layman users physically or cognitively. We evaluate *SCALING* via simulations and two testbeds (in lab and home configurations of sizes 3$\times$6 sq m and 4.5$\times$8.5 sq m). Experimental results demonstrate that *SCALING* outperformed the baseline using the approximate multidimensional scaling (MDS, the most relevant method in the context of self calibration) by 3.5 m/1.6 m in 80-percentile error of self calibration and tracking, respectively. Notably, only 1% degradation in performance has been observed with *SCALING* compared to the classical multilateration with known sensor locations (anchors), which costs hours of intensive calibrating effort. In addition, we conduct Monte Carlo experiments to numerically analyze the impact of sensor placements and develop practical guidelines for deployment in real life scenarios.

## Introduction

With rapid advancements in Internet of Things (IoT), in-home health monitoring systems are receiving increasing attention in our aging society with the goal of creating smart homes that support older adults to age in place. Among several types of monitoring data, users’ daily indoor trajectories are invaluable for health status assessment. With that, we can extract the low-level information about how capable or how fast a user can ambulate from one place to another. We can further derive the high-level information, for example, the duration and the frequency of engagements in certain functional spaces, such as the bathroom. Such information provides insights about users’ daily routines and behavior patterns, critical indicators not available from hospital visits (e.g., hesitant steps could indicate the onset or progression of Alzheimer’s dementia).

**Figure 1:**
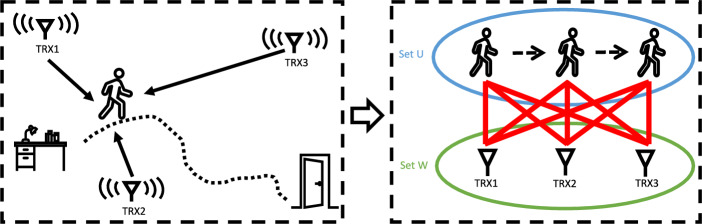
We present *SCALING* (Self-Calibrating Indoor Tracking), which uses distributed monostatic radars to measure distances to the human subject for multilateration thus tracking daily indoor trajectories, informative for health status assessment. We aim to save intensive efforts on calibrating sensor placements with a novel self calibrating algorithm which formulates the problem in a bipartite graph and obtain the uniqueness of geometrical topology according to the rigidity of graph. With that, the daily indoor trajectories can be extracted in this local positing system in the absence of anchors of known locations to be referenced.

### Prior work on device-free indoor tracking and scoping

Device-free schemes for indoor tracking are user-friendly because they do not require users to carry or wear any devices. Among typical categories, geophones or vibration sensors require costly refurbishment for large coverage^1,2^; vision^3^ or acoustic^4^ based monitoring solutions are highly susceptible to background interferences (lighting conditions or background noises), and sometimes may incur privacy concerns. Recent efforts have shown preferable features of RF techniques^5^, which are free of the aforementioned issues. People have explored localization/tracking using WiFi^6,7^, FMCW^8,9^, UWB^10,11^ based on proximity^12^, fingerprinting^13^, radio tomographic imaging^14,15^, parameter joint estimation^6,16^, and triangulation (including trilateration and angulation)^7^. While such solutions were reported with promising results, they usually rely on well-calibrated sensor placements, which require hours of intensive manual setup and respective expertise, feasible only at small scale and by mostly researchers themselves. Scaling the deployments to tens or hundreds of real homes, however, would incur prohibitive manual efforts, and become infeasible for layman users. Some may argue that RF sensing systems with single-site configurations^8,9,17^ can be preinstalled and wrapped up in a compact box thus easy for scaling. However, their localization performance will degrade significantly when the subject is far away from the single-site sensor due to the fixed angle resolution^7^, and coordinating their hand-off from one site to another remains an open challenge^9^. Therefore, we limit the scope of our paper to device-free indoor localization/tracking using distributed setups of multiple sensors, which by nature eliminate the hand-off concerns by consistent coverage thus localization performance no matter where the subject is located.

### Design considerations

With the above discussion, the costly sensor deployment effort for scaling remains as an issue to be solved. More specifically, the core challenge comes from the necessity of calibrating sensor placements with careful measurements and respective expertise. To address that, we present a plug-and-play indoor trajectory monitoring system that layman users can easily set up by walking a one-minute loop trajectory after placing sensor nodes on walls. For practical scaling purposes, we consider using low cost COTS RF devices in distributed nodes. To be specific, we use COTS monostatic radars^18^ in this study, each configured with a single pair of co-located transmitter and receiver. This radar facilitates interference-free simultaneous sensing by dithering phase for orthogonality^19^ at the cost of cross-talk between nodes. Therefore, the distributed nodes can only measure the distance of the target based on the channel impulse response^19^ but no pairwise distance measurement. While such configurations add to challenges from different levels, our design is without loss of generality because it works with minimum requirements on RF sensors and is applicable and compatible with more advanced RF devices (e.g., those with simultaneous communication and sensing) for self calibration.

### Our method

Taking into consideration the above challenges, we introduce a self calibrating algorithm that automatically estimates sensor locations using only distance measurements to the person walking in the monitoring space. The algorithm takes simultaneous observations from distributed nodes to optimize the estimation of relative sensor locations which minimize the distance measurement errors as an inverse process of multilateration, and we ensure its convergence at a unique solution by formulating it in a bipartite graph and analyzing its rigidity. Figure 1 shows the intuition of *SCALING* ($\underline{{\textbf {S}}}$elf-$\underline{{\textbf {Cal}}}$ibrating $\underline{{\textbf {In}}}$door Trackin$\underline{{\textbf {g}}}$). This self calibrating process only takes a trivial effort without taxing layman users physically or cognitively, and the average computing time is within one minute and can concurrently finish at the stop of one-minute walking trajectory in the online mode. We evaluate our design via simulations and two real-world testbeds, one with dense deployment in a lab of 3$\times$6 sq m and the other one with sparse deployment in a home configuration of sizes 4.5$\times$8.5 sq m. Volunteers with no expertise in calibrating sensor placements were invited for data collection, during which they walked freely in the monitoring space without specific instructions to be followed and even without knowing where the sensors were mounted. According to volunteers’ feedback, they all appreciated the easiness of the data collection process because they were not required to be trained or instructed to conduct walking freely, which only costed them about one minute on average. Experimental results demonstrate that our design achieves satisfying accuracy with the 80-percentile error of 53 cm in estimating the sensor locations and 40.5 cm in tracking the subjects, and largely outperforms a baseline with the adapted MDS, by 3.5 m/1.6 m in in 80-percentile error of self calibration and tracking, respectively. Notably, when comparing the tracking accuracy of self calibrated setup using our method against the classical multilateration with known sensor placements, we found that we can save hours of intensive calibrating effort with respective expertise to achieve comparable indoor tracking performance at the cost of only 1% degradation in accuracy, which is negligible. To develop practical guidelines for deployment in real world scenarios, we study the impact of sensor placements with numerical analysis. We conduct Monte Carlo experiments to simulate the impact of different sensors placements on the localization accuracy by carefully setting parameters to reflect practical measurements and reliable results from real-world experiments, while saving intensive efforts of repeating similar experiments on testbeds. The experimental results of the numerical analysis suggest that a configuration with widely distributed sensor placements, evenly or randomly surrounding the monitoring area, has consistently satisfactory performance ($\sim 0.1$ m accuracy) while co-linear arrays have inconsistent accuracy with an average of $\sim 2.2$ m.

### Overall contributions

We summarize our key contributions as follows:

- We propose *SCALING*, a plug-and-play device-free indoor trajectory monitoring system^20,21^ that a layman user can easily set up by walking a one-minute loop. It uses a self calibrating algorithm to eliminate the intensive manual efforts and technical expertise feasible with only researchers on calibrating the sensor placements (anchors) in prior work, paving the way for large scale self installation to benefit populations beyond small numbers of homes.
- We analyze self calibration of distributed sensor placements using only distance measurements in a bipartite graph, and ensure the uniqueness of its geometrical topology according to the graph rigidity. To achieve the convergence of self calibrating process, we introduce an iterative optimization in the mass-spring model^22^ using a sequence of walking trajectory to address the uncertainty in self calibrating sensor placements for indoor localization/tracking.
- We implement a prototype of *SCALING* using low cost COTS monostatic UWB radars and evaluate our design in two real-world testbeds, one with dense deployment in a lab of 3$\times$6 sq m and the other one with sparse deployment in a home configuration of 4.5$\times$8.5 sq m, in addition to extensive simulations. Experimental results demonstrate that *SCALING* achieves satisfying accuracy with the 80-percentile error of 53 cm in estimating the sensor locations and 40.5 cm in tracking the subjects, accurate enough to extract informative daily in-home trajectories. When comparing the tracking accuracy of self calibrated setup using our method against the classical multilateration with known sensor placements, the results imply that we can save hours of intensive calibrating effort with respective expertise at the cost of negligible degradation in accuracy by 1%.
- We study the impact of sensor placements both analytically and numerically. We compare the tracking/localization performance between different configurations of sensor placements through Monte Carlo experiments, in which we use a new metric to measure the global localization accuracy in an extended monitoring area rather than a single position, and introduce a greedy search algorithm to approach the quasi-optimal configuration of sensor placements with reduced computational complexity compared to the combinatorial search. The experimental results imply that the configurations of sensors widely placed surrounding the monitoring area provide $\sim 10$ times better accuracy than in co-linear arrays, and such observations are summarized into practical guidelines for real world deployment.

The remainder of this paper is organized as follows: We first discuss the related work. Next, we describe the system design of *SCALING*, in which a self calibrating algorithm is introduced as a key enabler followed by two other design components - distance measurements and multilateration localization. Then, we describe the experimental methodologies and results. Finally, we discuss the limitations and opportunities, and conclude our work.

## Related work

In this section, we discuss the relevant work in four categories: device-free monitoring, RF-based indoor tracking systems, self calibration methods, and sensor placements for localization.

### Device-free monitoring

The device-free schemes for indoor monitoring are popular because of their user-friendly nature because they do not require users to carry any device to be monitored^23,24^. In recent work, the concept of “structures as sensors”^25^ becomes popular, which aims to indirectly sense humans and surrounding environments through their structural responses by using geophones or vibration sensors. However, such systems usually require costly refurbishment for large coverage^1,2^. On the other hand, traditional methods using cameras with advanced computer vision methods^3,26^ are still useful for surveillance in the public space. Research also investigated acoustic sensors for in-home monitoring with prevalent IoT devices, e.g., Amazon Echo and Google Home^27,28^. However, either vision based surveillance^3^ or acoustic based monitoring^4^ solutions are highly susceptible to background interference (lighting conditions or background noises) and sometimes may incur privacy concerns. We choose to exploit RF-based techniques, which is free of the aforementioned issues.

### RF-based indoor tracking systems

Because of preferable features of RF-based solution, people have widely explored localization/tracking using different RF techniques. WiFi devices^6,7^ are popular for research investigation in this field because of its ubiquitous feature. With recent rapid advancements, more COTS RF devices become available, and people start to look into FMCW^8,9^ and UWB^10,11^ for indoor sensing because of their fine-grained ranging resolution. RF-based indoor tracking solutions have been extensively studied through different measurements and algorithms. With the available received signal strength index (RSSI), information related to the received signal strength (RSS), proximity-based algorithms have been proposed^12^. Furthermore, the mapping between RSS/RSSI and corresponding locations can be built and leveraged for indoor tracking through fingerprinting^13^. Besides, the radio tomographic imaging^29,30^ emerged as a promising device-free indoor tracking method based on the model of RSS of connected wireless networks. With more measurements of RF characteristics becoming accessible, the parameter joint estimation^6,16^ and triangulation (including trilateration and angulation)^7^ have been proposed for finer grained indoor tracking. While such solutions were reported with promising results, they usually rely on well-calibrated sensor placements, which require hours of intensive manual setup and respective expertise, feasible only at small scale and by mostly researchers themselves. Scaling the deployments to tens or hundreds of real homes, however, would incur prohibitive manual efforts, and become infeasible for layman users. Some may argue that RF sensing systems with single-site configurations^8,9,17,31^, can wrapped up in a compact box thus easy for scaling. However, their localization performance will degrade significantly when the subject is far away from the single-site sensors due to the fixed angle resolution^7^, and when extending to multi-site setup to address this issue, coordinating their hand-off from one site to another still remains an open challenge^9^. Therefore, we build our system using distributed setups, which by nature eliminate the aforementioned issues by consistent coverage thus localization performance no matter where the subject is located.

### Self calibration

Self calibration^32,33^ is always a hot topic in the field of sensor networks, because it is a key enabler and promising for large scale deployment. We have seen a good body of work in self calibration for indoor localization/tracking. Research exploited prior knowledge about the participatory walking trajectory for self calibration^34^ When the cross communication between neighbour nodes is available, Multidimensional Scaling (MDS)^35^ has been applied for self calibration^36^ given node-to-node distance measurements. Recent work leveraged MIMO platforms for self calibration, which is based on their satisfying space resolution to ensure the uniqueness of the pedestrian trajectory^37,38^. However, the existing work either leverages the prior knowledge about the moving trajectories, or based on advanced RF platform with additional information than pure distance measurements. To the best of our knowledge, we are first to investigate self calibration using monostatic radars of minimum requirements (with only distance measurements to the walking subjects available).

### Sensor placement for localization

The impact of sensor placements on the tracking/localization performance is well known and has been widely studied, especially in the communities of the Global Positioning System (GPS)^39,40^ and the Underwater Acoustic Positioning^41^. While RF-based indoor tracking^6,8,9,42^ becomes popular for smart home applications^43,44^ with the advances of IoT technologies, the optimization of sensor deployment in smart homes has not received sufficient attention and has been comparatively less investigated in the community of IoT and smart home sensing. Researchers have recognized the Information Inequality^45^ as an important means of analyzing and optimizing localization accuracy based on the configuration of sensor placements and the characteristics of direct measurements (e.g., distances and/or angles). There are further efforts on investigating optimal sensor placements for tracking targets^46–48^ under specific constraints (e.g., along a predefined path). However, there exists no analytical expression to derive optimal sensor placements for localization in an extended monitoring area rather than a single position. Following two recent works^49,50^ on finding the optimal sensor placements with numerical analysis, we conduct Monte Carlo experiments to study the impact of sensor placements and develop guidelines for sensor deployment in practical scenarios. While the uncertainties in sensor locations due to self calibration error may introduce additional impact on tracking/localization performance, it has been solved using the constrained least square technique (CTLS) with a derived Cramer-Rao low bound (CRLB)^51–53^, and is out of the scope of this study.

### Ethical approval

The study protocols involving data collection with human subjects were reviewed and approved by the IRB committee of Stony Brook University (IRB2021-00521), and informed consent was obtained from all participants.

## System design of SCALING

In this section, we introduce *SCALING* ($\underline{{\textbf {S}}}$elf-$\underline{{\textbf {Cal}}}$ibrating $\underline{{\textbf {In}}}$door Trackin$\underline{{\textbf {g}}}$), a plug-and-play device-free indoor trajectory monitoring system that a layman user can easily set up by walking a 1-minute loop, thus free of the intensive manual efforts and respective expertise. Our system uses distributed monostatic radars to collect concurrent distance measurements for localization. The key enabler of our system is a novel self calibrating algorithm that estimates sensor locations based purely on distance measurements to the person walking a sequence of unspecified trajectories in the monitoring space. Upon the convergence of self calibrating process, we use the estimated sensor locations as pseudo anchors for multilateration, different from the existing work using know sensor locations.

**Figure 2:**
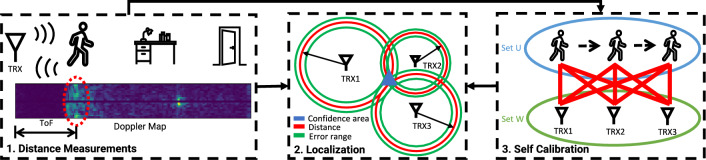
The overall framework of *SCALING* ($\underline{{\textbf {S}}}$elf-$\underline{{\textbf {Cal}}}$ibrating $\underline{{\textbf {In}}}$door Trackin$\underline{{\textbf {g}}}$), which has three major components: 1. The human subject is detected for **distance measurements** in cluttered environment through the Doppler Map. 2. Simultaneous distance measurements from distributed nodes are fed to the multilateration algorithm for **localization**, given known sensor locations. 3. A novel **self calibrating** algorithm formulates the problem in a bipartite graph and leverages its rigidity to obtain the uniqueness of geometrical topology for tracking trajectories of a walking person in the self calibrated local coordinate. With the first two components, we can build a device-free tracking system at cost of intensive manual effort and respective expertise on calibrating sensor placements. The third component estimates the relative sensor locations automatically, thus eliminating the need for manual calibration.

Figure 2 shows the overall framework of *SCALING*, which has three major components:

- Self Calibration: We first introduce “Self Calibration” as the key enabler of *SCALING*. When the locations of distributed nodes are unknown and the only available information is the distance measurements to the human subject walking unspecified trajectories in the monitoring area, we form the problem in a bipartite graph, of which the edges are the distance measurements between the unknown sensor locations and unknown participatory step locations in walking trajectories. With only distance measurements as constraint, the vertices may flex thus varying the geometrical topology of the bipartite graph. With the analysis of graph rigidity to ensure the uniqueness of the geometrical topology, we use iterative optimization to reach convergence of estimated sensor locations.
- Distance Measurements: Distance measurements are non-trivial. Each distributed monostatic radar detects the human subject from cluttered home environment and measures their distance according to ToF of the emitted signal bounced off the human body back to the corresponding receiver.
- Localization: We apply multilateration algorithm for localizing thus tracking the subject when the distance measurements of a subject in the monitoring area are available from distributed nodes, assuming the locations of distributed nodes are known. We also analyze the impact of sensor placements.

We start with discussion of design considerations to help shape the problem, and then detail the design components.

### Design considerations

Out of practical considerations for future large scale deployment of device-free indoor tracking systems, we have to balance trade-offs among several key factors: localization/tracking performance, deployment efforts on well-calibrated sensor placements, and cost of RF sensor nodes. Distributed configurations provide consistent localization/tracking performance in contrast to single-site configurations, at the price of prohibitive deployment cost. Therefore, we are challenged to minimize the deployment cost, which mainly comes from intensive efforts in calibrating sensor placements with careful measurements and respective expertise, in addition to the expense of sensor nodes.

**Hardware choices** While we know the goal of hardware choices is to minimize the expense of sensor nodes, it is non-trivial to understand what it takes to meet the minimum requirements for device-free indoor tracking:

- For the minimum overhead on communication, we consider the case that sensor node will only forward measurement data to the central server for computations, but there is no cross-talk among sensor nodes to exchange information.
- Each node has to be configured with at least one transceiver so that multilateration can be applied for localization with distance measurements from distributed nodes.
- The critical but tricky part is that we have to ensure the orthogonality of RF signals for simultaneous sensing with distributed nodes, when the coordinated transmission is unavailable with no cross-talk.

To address the above concerns all together, we use a low cost COTS UWB sensor^18^ which achieves orthogonality by dithering phase^19^, such that concurrently emitted signal will not interfere each other even in the absence of cross-talk to coordinate transmission among distributed nodes. This COTS UWB sensor is a monostatic radar, configured with a single pair of co-located transmitter and receiver. It measures the distance of the target according to the time of flight (ToF) of the repeatedly emitted signal bounced off from the target and captured by the receiver. Herein, we clarify our hardware choices to meet minimum requirements for device-free indoor tracking.

**Design goals** The remaining issue to minimizing the deployment cost is to mitigate the intensive efforts in calibrating sensor placements with careful measurements and respective expertise. That unveils the major goal of this paper, which is to design a plug-and-play device-free indoor tracking system using low cost COTS RF devices. And the key enabler of this system is a novel self calibrating algorithm, that eliminates the need for intensive manual calibrating effort on sensor deployment. While this design goal is derived from the configuration of minimum requirements premised on specific hardware choices, we believe the discussion of such design is without loss of generality, because it works with RF sensors of minimum requirements and is applicable and compatible with more advanced RF devices for self calibration^37,38^. We will clarify how our design can be applied to advanced RF devices given more features in Discussion section.

### Self calibration

This section introduces a novel self calibrating algorithm that estimates the relative sensor locations through purely the distance measurements to walking trajectories of the human subject in the monitoring area by leveraging the rigidity of bipartite graph. This estimated sensor locations are used as anchors for localization/tracking together with the previous two design components. The proposed self calibrating algorithm largely reduces hours of intensive calibrating efforts with respective expertise to merely one-minute walking affordable by layman users at cost of negligible loss in localization/tracking accuracy.

We first describe our self calibrating algorithm that estimates the relative sensor locations using merely distance measurements to the walking trajectories. We also describe a relevant method and discuss why it is not applicable in this problem, and how we adapt it as a supplementary process.

#### Self calibrating algorithm

Consider *M* sensor nodes distributed at unknown locations in the monitoring space. As described in Sect. “Design considerations”, we assume each node can only measure the distance to the present human subject, but not node-to-node pairwise distance because their signals are orthogonal thus no cross-talk. Distance measurements are available for *N* step locations in the walking trajectories of the human subject. The task is formed as to estimate the relative sensor locations using distance measurements only. For clarity, we assume the following discussion of localization/tracking is in 2D plane (${\mathbb {R}} ^2$) if not specified; the 3D version is a simple and natural extension.

**Figure 3:**
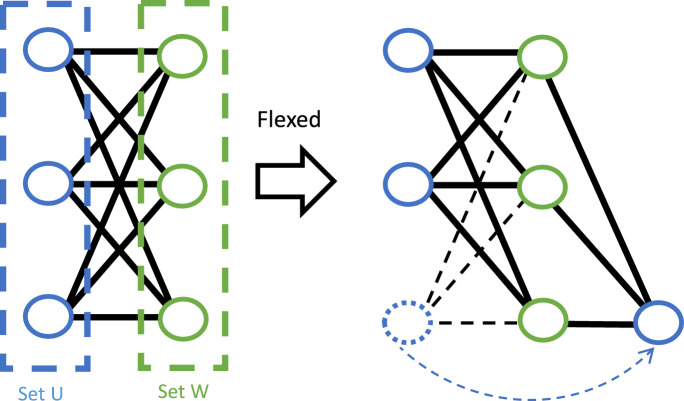
In this example graph, a vertex in Set *U* could flex while preserving the distances.

We first analyze whether it is a solvable problem and in what conditions. When the problem is modeled in a linear system, a natural but naive way is to check whether it has more independent equations of constraints/observations than the number of unknowns. However, our problem is modeled with constraints from distance measurements, which is quadratic but not linear, so the necessary and sufficient conditions for solvability of linear systems are not applicable here. We formulate the problem in a graph *G*(*V*, *E*), as illustrated in the third part of Fig. 2, to determine whether it is solvable to estimate relative sensor locations by analyzing the uniqueness of the geometrical topology. *V* denotes the vertices, including *M* sensor locations (denoted as $a_i \in W$) and *N* step locations (denoted as $p_j \in U$) in walking trajectories where the distance measurements to the human subject are taken. *E* denotes the edges, corresponding to the distance measurements $d_ij$ between the sensor locations $a_i$ and step locations $p_j$. Apparently, this graph is a bipartite graph and is a complete bipartite graph because there is no edge among the subsets of sensor locations *W* or step locations *U*, every edge in *G*(*V*, *E*) connects a vertex in *W* to one in *U*, and every pair of vertices between Set *W* and Set *U* are connected.

We note that the coordinate assignments thus the geometrical topology of the bipartite graph may not be unique even up to rotation, translation, and reflection, because certain vertices could flex while preserving the distances, as illustrated in Fig. 3. To obtain a unique geometrical topology, the graph has to be globally rigid^54,55^. According to characteristics of the globally rigid graph^56,57^, several conditions need to be met:

1. The distribution of vertices (including sensor locations and step locations) needs to be generic, thus no three vertices form a line like the distribution of Set W in Fig. 3.
2. Applying Euler’s formula gives the relation between the number of edges *l* and the number of vertices *m*, $l \ge 2m - 3$. In our case, $l=M\times N$, $m=M+N$, so we get $M\ge 2+\frac{1}{N-2}$ and $N\ge 2+\frac{1}{M-2}$.
3. Globally rigid graph needs vertices to be (d+1)-connected in ${\mathbb {R}}^d$, thus 3-connected in 2D, met by nature of the complete bipartite graph.

The last two conditions can be easily met when we use least three sensor nodes and three step locations. For the first condition, we can distribute sensor nodes to different walls around the monitoring space to avoid forming a line. Although step locations in walking trajectories may fail to meet the first condition due to randomness, however, people usually would not walk along a perfectly straight line in natural settings. By randomly selecting step locations from a walking trajectory, it is rare that three step locations form a line and can be mitigated with iterative optimization. To properly select step locations for self calibration in this implementation, We empirically use a time interval of 5 seconds to guarantee an adequate level of randomness in sampling step locations in walking trajectories. Notably, the case of three points on the same line can be translated to that the area formed by the triangle of these three points is equal to zero. For simplicity of illustration, we denote three sides of the triangle *a*, *b*, and *c*, and $s=(a+b+c)/2$, so the area of the triangle is expressed as $Area = \sqrt{s(s-a)(s-b)(s-c)}$. Then we use the unit-less measurement $\gamma =(Area/s^2)$ to indicate how perfectly a straight line might be formed by the three selected step locations, as the unit-less measurement $\gamma$ near zero means the three points nearly form a straight line, while an equilateral triangle gives $\gamma =0.1925$ on the other end. We observe that the distribution of $\gamma$ from our selected three step locations is characterized by a mean of 0.0794 and standard deviation of 0.0592. This suggests that our empirical selection makes it improbable for three step locations to align on the same line. We admit that the optimized selection of step locations is crucial to self calibration, however, it is beyond the scope of this paper.

Now we are confident about the solvability of our problem, and can focus on estimating the sensor locations through distance measurements to the step locations in walking trajectories. With the graph, our goal is to generate a geometrical topology in which the coordinate assignments of sensor locations $a_i \in W$ and step locations $p_j \in U$ are consistent with all distance measurements $d_{ij}$, $\left\| a_{i}-p_{j}\right\| =d_{i j}$ for all $d_{ij} \in G(V, E)$. Therefore, we can estimate of sensor locations by optimizing each residual between the measured distance $d_{i j}$ and the estimated distance (between the estimated sensor location $a_i$ and the corresponding step location $p_j$), defined as $e=|\Vert a_{i}-p_{j}\Vert - d_{i j}|$. We adapt it in the mass-spring model for optimization, where each edge in the graph is taken as a spring between two masses, with a rest length equal to the measured distance. When the estimated distance between $a_i$ and $p_j$ is shorter than the measured distance, the spring incurs a force that pushes two nodes apart. Similarly, when the estimated distance is larger than the measured distance, the spring pulls them together. Along the optimization process, the estimated nodes move in the direction of the resulting force of the spring.

The stress of this mass spring model to be optimized is expressed as:

1 $$\begin{aligned} \text { Stress }=\frac{\sum \left( d_{i j}-\Vert a_{i}-p_{j}\Vert \right) ^{2}}{\sum \Vert a_{i}-p_{j}\Vert ^{2}} \end{aligned}$$

When the stress becomes zero, the whole mass-spring system reaches equilibrium, so the optimization process reaches the global minimum. We empirically choose Sequential Least Squares Programming (SLSQP) as the optimization solver. Because the measured distance is noisy, the estimated sensor locations are expected to jiggle to a certain extent. Instead of using one-time estimated sensor locations *W*, we combine results from multiple rounds. At the k-th round, we select a set of step locations along walking trajectories (denoted as $U_k$) with corresponding distance measurements to estimate the configuration of sensor locations (denoted as $W_k$). A new round of self calibrating process will be executed with a new set of step locations $U_{k+1}$ until the residual difference between $W_k$ and $W_{k-1}$ reaches a small empirical threshold.

#### Approximate MDS for initial coordinate assignment

While the mass-spring model is powerful for self calibrating process, it has a chance to converge at local minimum if it starts with a random initial coordinate assignment. We aim to introduce a supplementary process that provides the self calibrating process with a reliable initial coordinate assignment with a similar geometrical topology to the ground truth, such that the mass-spring model can easily converge at a global minimum rather than a local minimum. One relevant method, Multidimensional Scaling (MDS)^35^, is often used as a dimension reduction technique to graphically visualize data in 2D, and can be applied to convert distance measurements between nodes into node locations^58^.

**Figure 4:**
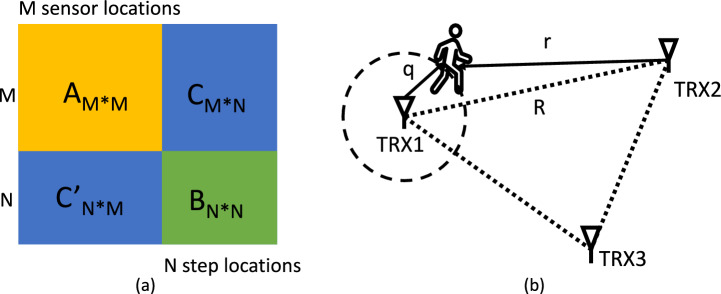
Approximate MDS. (**a**) Distance matrix. The sub-matrix A/B denotes the pairwise distance measurements within sensor/step locations (Set *W*/*U*). Sub-matrices C and C’ denote distance measurements across sensor locations Set *W* and step locations Set *U*. (**b**) The step location close to a certain sensor location opens the opportunity to approximate the sensor-to-sensor distance measurements with sensor-to-step distance measurements for MDS to achieve initial topology. While distance measurements are only available in C and C’, we can use sensor-to-step distance measurements to approximate sensor-to-sensor distance measurements. Therefore, we can use MDS to achieve an initial coordinate assignment with an approximate sensor-to-sensor distance matrix A’.

While MDS requires a complete set of node-to-node distance measurements, it is not available in our problem as illustrated in Fig. 4a: we only have available distance measurements across sensor locations Set *W* and step locations Set *U* in sub-matrices C and C’, but the pairwise distance measurements within Set *W* or Set *U* corresponding to sub-matrices A or B are not available. The fact that certain step locations in random trajectories are close to a certain sensor location opens the opportunity for using MDS with approximate node-to-node distance measurements for initialization. As shown in Fig. 4b, given a step location close to a certain sensor node 1, we obtain the triangle inequalities: $r-q<R<r+q$. According to the Squeeze Theorem, we can use the sensor-to-step distance *r* to approximate the sensor-to-sensor distance *R* when *q* is relatively small. Notably, because MDS requires the distance matrix to be positive semi-definite, we keep the elements symmetric to the diagonal by using the one closer to the sensor among a pair. Therefore, we can use MDS with the approximate sensor-to-sensor distance matrix A’ for initialization of coordinate assignments.

**Figure 5:**
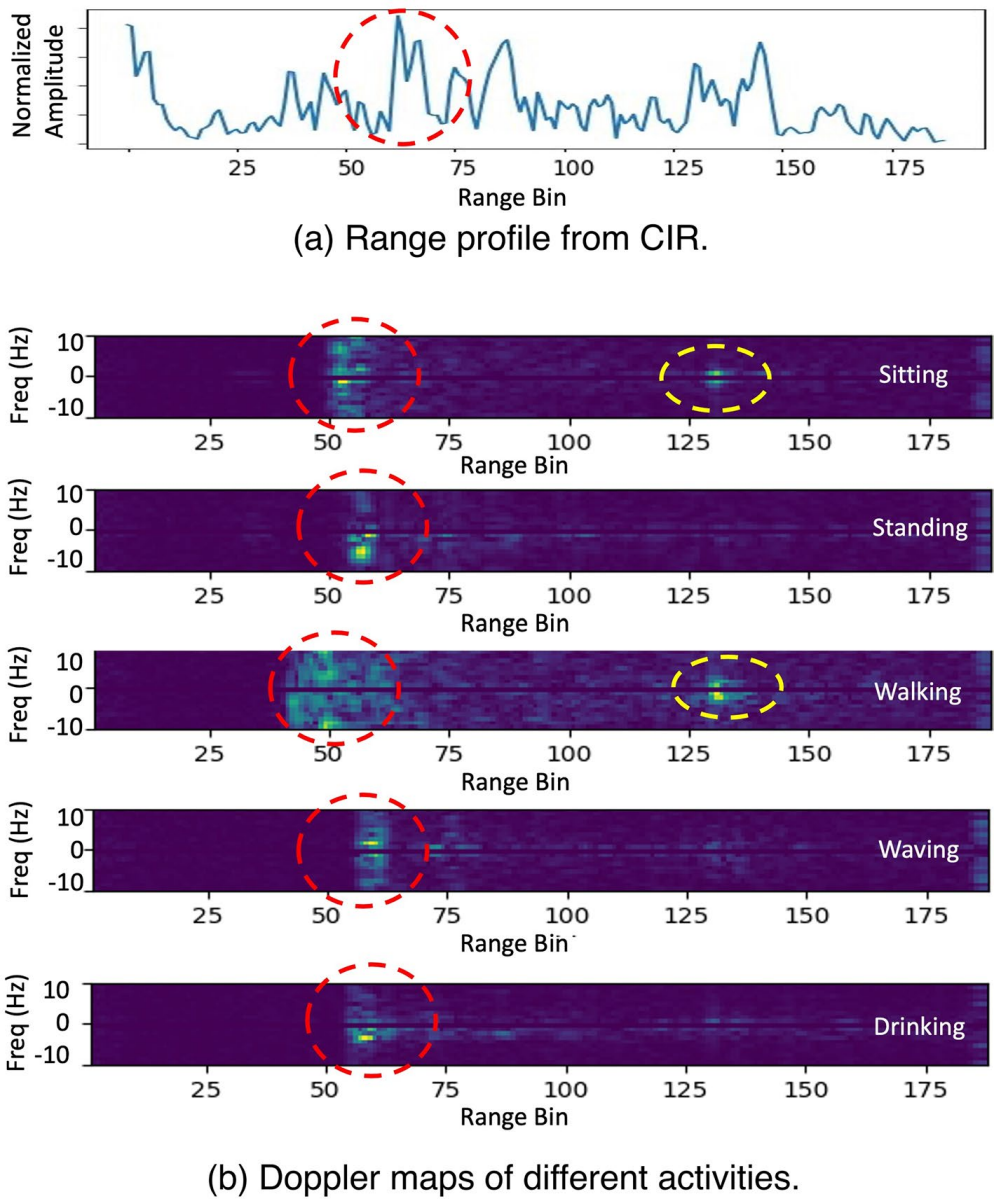
(**a**) shows a range profile, where the channel impulse response due the human subject is not easily distinguishable. (**b**) shows Doppler maps of different activities. The x-axis of the Doppler map is the index of range bins, corresponding to the distances aligned with the range profile; y-axis shows the index of frequency band determined by the length of time window and the frame rate of the range profiles. The color code of the plot corresponds to a heat-map of the intensity in the reflected signal. Strong reflectors are indicated by light colors such as yellow and green, weaker reflectors are indicated by dark blue, and the absence of a reflector is indicated by black at the corresponding frequency. The red circles indicate the reflection from a human subject, and the yellow circles indicate the multi-path effects.

### Distance measurement

As described in Sect. “Design considerations”, we use low cost COTS UWB radars at the band 7.25–10.2 GHz in distributed nodes for distance measurements, and this UWB radar was designed with swept-threshold sampling with cumulative 1-bit quantized value^19^, enabling a high-speed sampler that operates at 23.328 GS/s, sufficient to sample received signals at high resolution. With that, it may appear to be straightforward to obtain distance measurements to the human subject as the time of flight (ToF) can be estimated according to the delay between transmission of short UWB pulses and receiving the signals reflected by the human body. However, two key factors challenge reliable distance measurements: (1) The multi-path effect may cause ambiguity in differentiating the human subject from the static objects in the cluttered environment. (2) The human body is more complex than a point scatterer, so echoes may come from the head, arms, legs, and torso, which span dozens of centimeters in space, introducing ambiguity in determining the actual distance between the human subject and sensor nodes.

To localize human subjects, we first have to detect them from the cluttered environment, so we need to eliminate the impact from static reflectors. Figure 5a shows the range profile generated directly sampled Channel Impulse Response (CIR), from which the reflection from human body is not prominent for detection. The x-axis of the range profile is the index of range bins, linearly proportional to the distance; y-axis shows the normalized amplitude of the received signal; a point in the curve of the range profile indicate the intensity of the reflection from a certain distance. We detect the human subject by leveraging the observation that signals from static reflectors are constant in both amplitude and phase over time, different from Doppler effects caused by moving subjects.

We visualize the Doppler effects^59^ in the reflected signal with Doppler maps^60^ by applying Short-Time Fourier Transform to a sequence of consecutive range profiles along the time dimension. Figure 5b shows five Doppler map examples, of which the data was collected when performing five typical indoor activities. While the patterns of Doppler maps may differ between different activities, the moving human subject is always highlighted with prominent intensity in the Doppler map.

After eliminating static components, we notice that the signals reflected from human body may spread thin in a wide chunk because different body parts move differently and the corresponding reflections may interfere each other constructively or destructively. Besides, the Doppler map may show the highest intensity at a distance other than where the human subject is located due to dynamic multi-path components^8^, which are the reflections that bounce off the human body then bounce off static objects in the cluttered environment before arriving at the receiver. To reduce the ambiguity from the dynamic multi-path components, we reject them based on that fact that dynamic multi-path signals are always traveling longer paths and coming later than the direct reflections from the human body. To be specific, we apply CFAR^61^ to detect pixels in the Doppler map corresponding to the valid reflections and take the closest chunk of detected pixels as the reflections from human body according, and the distance measurement to the human subject is calculated as the distance to the mass centroid of the closest chunk:

2 $$\begin{aligned} d_{mc}=\lim _{\Delta m \rightarrow 0} \frac{\sum _{i=1}^{N}\sum _{j=1}^{M} \Delta m_{ij} d_{j}}{\sum _{i=1}^{N}\sum _{j=1}^{M} \Delta m_{ij}}, \end{aligned}$$

where *i* is the index of total *N* frequency bands, *j* is the index of total *M* range bins, $\Delta m_{ij}$ is the intensity of the pixel at the index of (i, j) in the Doppler map, and $d_{j}$ is the corresponding distance of the range bin of index *j*.

### Localization

As discussed in Sect. “Design considerations”, we aim to develop a device-free tracking system using low cost RF sensors of minimum requirements thus without loss of generality. Therefore, the only available information for localization is the distance measurements through the time of flight, but the angle of arrival is not available due to the limited space diversity of the monostatic radar configured with a single pair of co-located transmitter with receiver. To achieve angle information with limited space diversity, some existing work^31^ explored a classical radar technique called inverse synthetic aperture radar (ISAR)^62^, which emulates a virtual antenna array in the reference system of the moving target. While this method was able to detect sharp changes in the angle, it is not applicable for fine-grained trajectory tracking, because the angle resolution of ISAR methods, determined by the time window of observations, is relatively low and not sufficient to capture the walking movement of low speed and changing direction. The coarse-grained angle information from ISAR may add some constraints for localization through joint parameter estimation, it is out of the scope of this paper. We will focus on the discussion of using distance measurements only for localization. After detecting the human subject in the cluttered environment by addressing ambiguities from multi-path effects with the Doppler map, each sensor node obtain reliable distance measurements to the human subject. Then, we can combine simultaneous distance measurements from distributed nodes for localization. Given known sensor locations, we can easily estimate the location of the human subject which gives the least squared error of distance according to their respective distance measurements.

**Figure 6:**
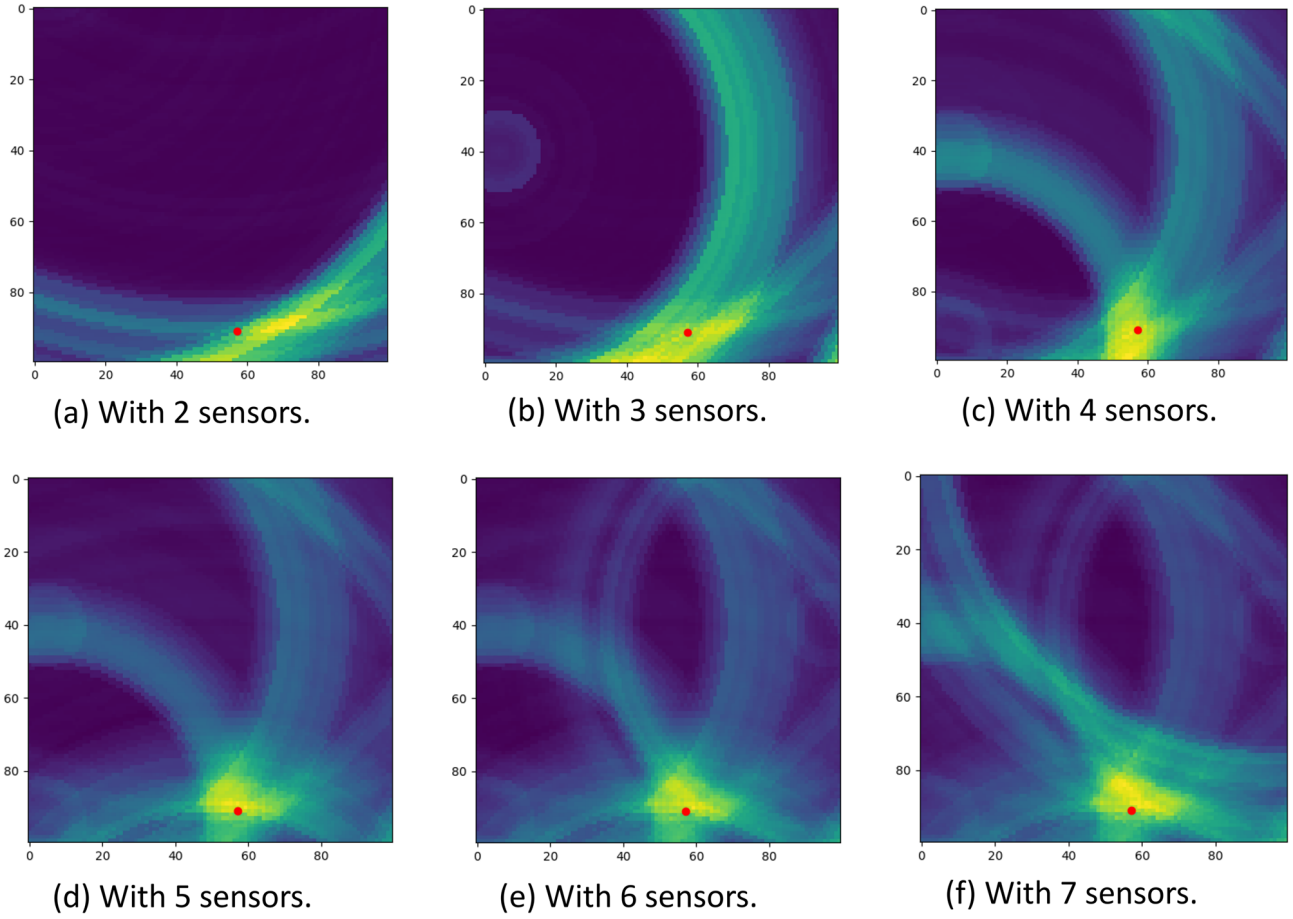
We use different number of sensors to localize a target in a 1m$\times$1m space. The red dot indicates the ground truth of the human subject’s location. With increasing number of sensor nodes, the intersection region is with less uncertainty, thus more accurate.

For simplicity, we illustrate localization in 2D plane as shown in the middle part of Fig. 2. When we consider just one transceiver, one distance measurement can form one circle around the sensor location, indicating the confidence area where the human subject is located assuming no other constraints applied. At any time, if there are at least three distributed sensor nodes available for simultaneous distance measurements, we can find a intersection between three circles to locate the human subject. To generalize the argument to localization in 3D space, we only need one additional sensor node. While three distributed sensor nodes are sufficient for getting a unique estimate of the human subject’s location in 2D plane, we notice that increasing the number of sensor nodes can help reduce the uncertainty in localization with noisy distance measurements. Figure 6 shows the uncertainty of localization can be reduced with increasing number of sensor nodes. We will study the impact of the number of sensor nodes on our design in Evaluation section.

To formally represent and analyze the multilateration localization in a 2D space, we describe the mathematical model in the following. We denote the unknown coordinates of the human subject to be localized as $p=[x,y]^{T}$, and the coordinates of the *i*th sensor node as $a_{i}=[u_i,v_i]^{T}$. Given the estimate of the human subject location $p=[x,y]^{T}$, we express the hypothetical distance as:

3 $$\begin{aligned} g_i(p)=\left\| a_{i}-p\right\| =\sqrt{\left( x-u_i\right) ^2+\left( y-v_i\right) ^2}. \end{aligned}$$

The observations, i.e., actual distance measurements from the *i*th sensor node to the human subject are expressed as:

4 $$\begin{aligned} d_i = g_i({\dot{p}}) + \varepsilon , \end{aligned}$$

where ${\dot{p}}$ is the true coordinate of the human location, and $\epsilon$ is the additive error with a known covariance matrix determined by the actual distance measurements. With *M* sensor nodes, we define ${\underline{g}}(p)=[g_1({p}), g_2({p}), ..., g_M({p})]^T$, ${\underline{d}}=[d_1, d_2, ..., d_M]^T.$

The multilateration localization is to estimate *p* given $\{a_i, d_i; i=1,2,...,M\}$ by minimizing the the sum of squared residual errors between the measured distances $d_i$ and hypothetical distances $g_{i}(p)$, using, e.g., a least-squares estimator:

5 $$\begin{aligned} {\hat{p}}=\underset{p}{\arg \min }\left\{ \sum _{i=1}^M\left( g_i(p)-d_i\right) ^2\right\} =\underset{p}{\arg \min }\left\{ ({\underline{g}}(p)-{\underline{d}})^{T}({\underline{g}}(p)-{\underline{d}})\right\} . \end{aligned}$$

**Sensor placement.** We get a localization error when estimating the human location *p* using multilateration with distance measurements $d_i$ because of the measurement error $\epsilon$. We derive the relationship between measurement error and localization error:

6 $$\begin{aligned} {\text {C}}^{\epsilon } = {\text {H}}\ {\text {C}}^{p}\ {\text {H}}^{\top }, \end{aligned}$$

where ${\text {C}}^{\epsilon }$ denotes variance-covariance of distance measurement error $\epsilon$, and ${\text {C}}^{p}$ the variance-covariance of localization error. H is the Jacobian matrix for observations from all sensor nodes according to the gradient of the measurement model (4), expressed as:

7 $$\begin{aligned} {\textbf{H}}=\frac{\partial {\underline{g}}}{\partial p}= \left[ \begin{array}{c} \nabla g_1^{\top } \\ \vdots \\ \nabla g_M^{\top } \end{array}\right] =\left[ \begin{array}{ccc} \frac{p^{(x)}-a_1^{(x)}}{\left\| {\underline{p}}-{\underline{a}}_1\right\| } &{} \frac{p^{(y)}-a_1^{(y)}}{\left\| {\underline{p}}-{\underline{a}}_1\right\| } \\ \frac{p^{(x)}-a_2^{(x)}}{\left\| {\underline{p}}-{\underline{a}}_2\right\| } &{} \frac{p^{(y)}-a_2^{(y)}}{\left\| {\underline{p}}-{\underline{a}}_2\right\| } \\ \vdots &{} \vdots \\ \frac{p^{(x)}-a_M^{(x)}}{\left\| {\underline{p}}-{\underline{a}}_M\right\| } &{} \frac{p^{(y)}-a_M^{(y)}}{\left\| {\underline{p}}-{\underline{a}}_M\right\| } \end{array}\right] . \end{aligned}$$

Based on (6), we can easily derive

8 $$\begin{aligned} {\text {C}}^{p} = {\text {H}}^{\top }\ {\text {C}}^{\epsilon }\ {\text {H}}. \end{aligned}$$

Given a fixed measurement error $\epsilon$, the localization error can be larger or smaller depending on where the sensor nodes and the target human subject are situated in relation to one another. We are interested to reach a sensor placement that minimize the localization error. The Cramer-Rao bound of the variance-covariance ${\text {C}}^{\epsilon }$ is expressed in the information inequality as:

9 $$\begin{aligned} {\text {C}}^{\epsilon } \ge J_\epsilon ^{-1}, \end{aligned}$$

where $J_\epsilon$ is the Fisher information matrix for distance measurements ${\underline{d}}$, and is predetermined upon the devised algorithm for distance measurements. Therefore, we can improve the upper bound on the precision (i.e., the inverse of variance) of the location estimator by adjusting sensor placements, thus the Jacobian *H*. Note that *H* consists of direction vectors, each of which is a unit vector representing the relative direction from the sensor node $a_i$ to the human location *p*. For a specific *p*, the Jacobian *H* can be analytically optimized to minimize the variance-covariance $C^p$. An optimal configuration of sensor placements has been derived in the existing work^45,48^, which suggest a uniform spreading of sensor nodes around the target location *p*. Specifically, the angles between the two direction vectors (in Jacobian *H*) of two arbitrary neighboring sensor nodes are equal. However, in the real life scenarios, the target location is unknown ahead of time and is even dynamic relative to sensor placements. Therefore, the derived optimal sensor placements for a specific target location *p* may not hold in practical scenarios. Instead of identifying the configuration of sensor placements optimal to a specific target location *p*, we aim to find a globally optimal configuration that minimizes the average localization error in the whole covered area. While we know the localization accuracy are related to the sensor placements, a closed-form expression is not available for the theoretical analysis of the globally optimal configuration because it varies depending on the covered area. To achieve that, we conduct the numerical analysis (detailed in Evaluation section) through Monte Carlo experiments, based on which we develop some guidelines for practical sensor deployments.

## Evaluation

To evaluate our design, we conducted extensive experiments via two real-world testbeds as well as simulations. We first describe the experimental setups, the purpose experiments we conducted and the metrics and ground truth used for evaluation. Then, the experimental results with real-world testbeds are presented. We also conduct extensive simulations to study the impact of different factors on the end-to-end performance. In addition, we conduct numerical analysis to study the impact of sensor placements with Monte Carlo experiments, from which we develop practical guidelines for sensor deployment in real world scenarios.

**Figure 7:**
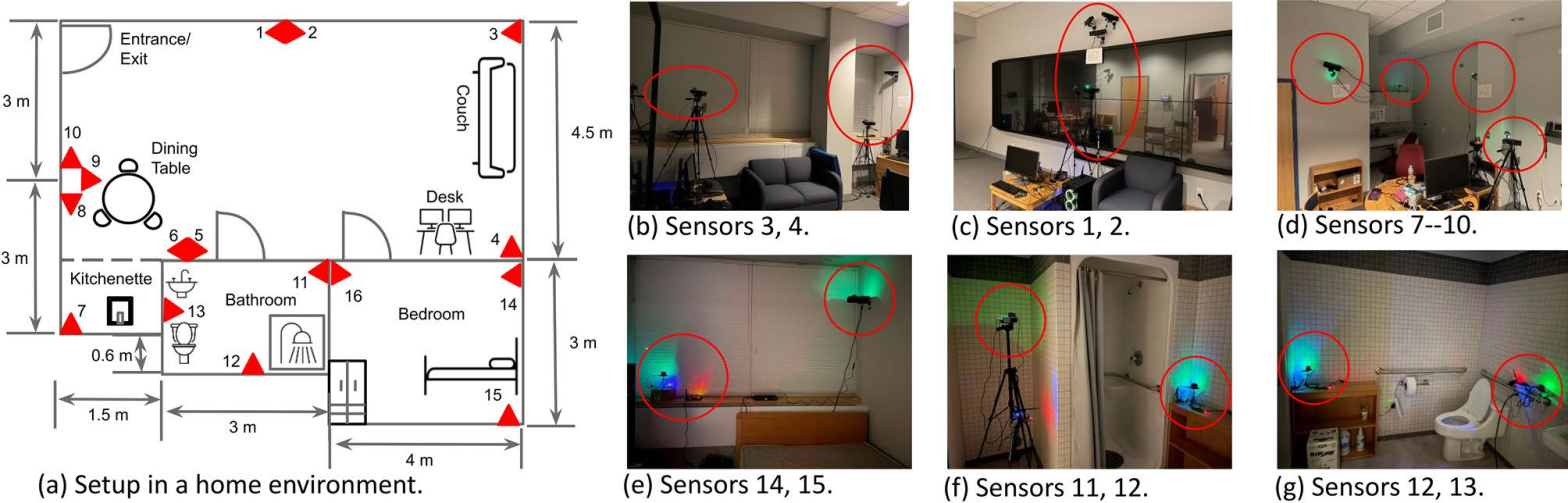
The testbed with sparse deployment in a cluttered home environment decorated with furniture.

**Figure 8:**
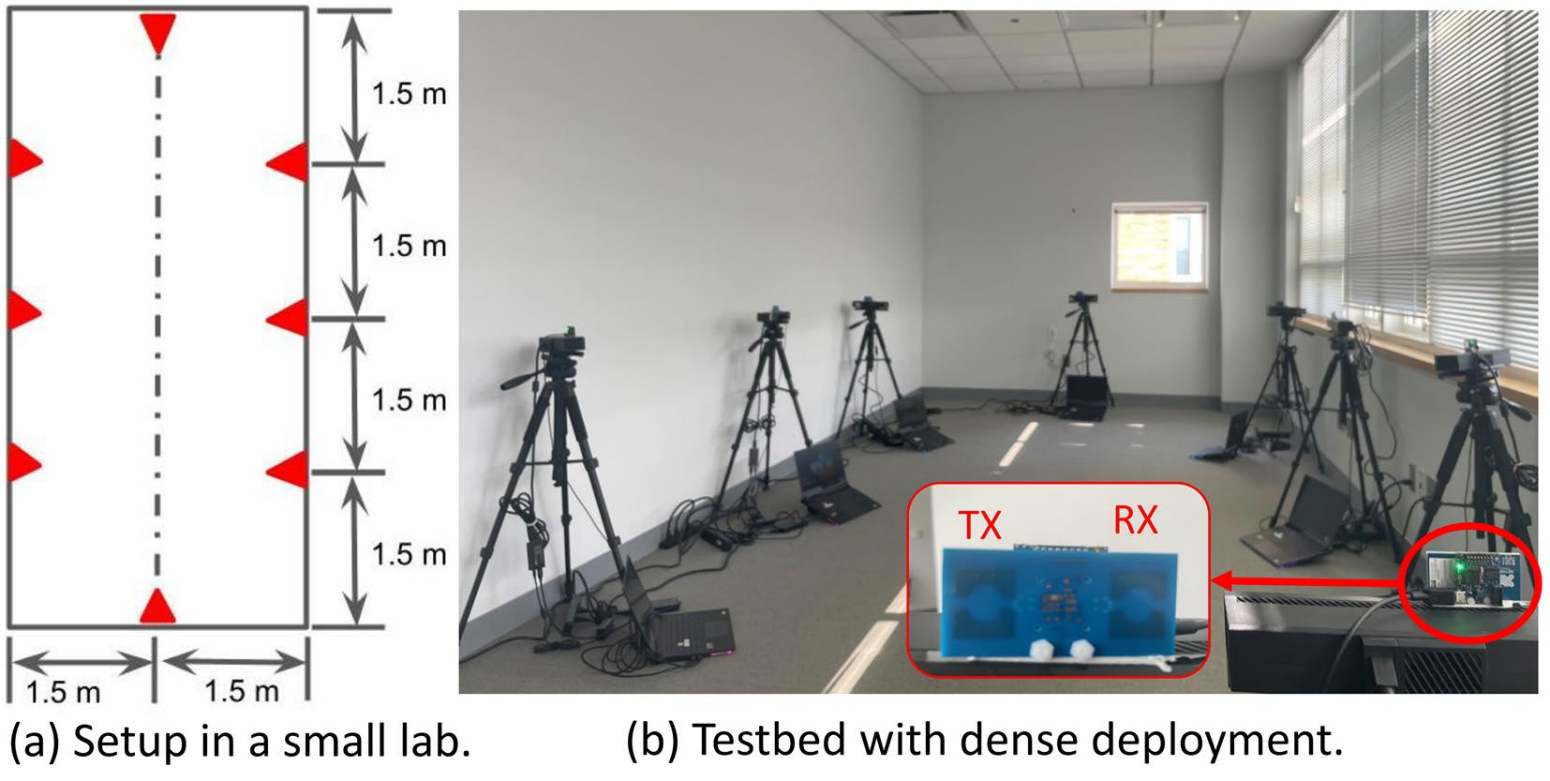
The testbed with dense deployment in a lab environment.

### Experimental methodologies

To collect real-world data for evaluation, we built two testbeds, one with sparse deployment in a cluttered home environment of size 4.5$\times$8.5 sq m, shown in Fig. 7, and the other one with dense deployment in an empty lab environment of 3$\times$6 sq m, shown in Fig. 8. Notably, settings in two testbeds configured with redundant sensors on tripods are for research purpose only. A much smaller number of sensors (e.g., mounted on walls) would be sufficient in real-world scenarios. The UWB signal can sense human movements through the wall/door with penetration ability^17^, thus can be shared across different rooms to further reduce the number of necessary sensor nodes. In each sensor node, we use a COTS IR-UWB sensor XeThru x4m03^18^ as a monostatic radar for distance measurements. The emitted pulse is configured with the frequency band 7.25–10.2 GHz centered at 8.75 GHz, and the sampling frequency of this COTS UWB sensor is 23.328 GHz, sufficient to capture reflections with a high resolution. The frame rate of the UWB sensor is configured to be 20 frame-per-second (fps), thus able to update distance measurements 20 times per second, and its measurement range is up to 10 meters. Distributed sensor nodes are synchronized through the Network Time Protocol (NTP), and concurrent sensing data (e.g., distance measurements) are forwarded to an on-site central server in batches and aligned according to the associated timestamps at a resolution of 0.05 sec for further computation (e.g., self calibration and localization/tracking) in a retrospective manner.

#### Data collection and experiments

The study protocols involving data collection with human subjects were reviewed and approved by the IRB committee of Stony Brook University (IRB2021-00521), and informed consent was obtained from all participants. During data collection, we strictly follow the established protocols and guidelines. We invited volunteers with no expertise in calibrating sensor placements, and they walked freely in the monitoring space without specific instructions and without knowing the sensor locations to generate random walking trajectories thus emulating the natural indoor trajectories as users will be performing in a real world scenario. According to the feedback from volunteers, they were not feeling challenged physically or cognitively during data collection, because it was as simple as taking a walk in a room for about one minute, even though they did not take any training program to prepare them for that. During data collection, we follow a pre-established protocol that protected the anonymity of the students. We use 50% of collected data for evaluation of self calibrating algorithm and the remaining 50% for evaluation of end-to-end tracking performance using the self calibrated sensor locations. We also use the approximate MDS as a baseline for comparison and compare the end-to-end performance with and without the approximate MDS for initial coordinate assignments. In addition, we conducted extensive simulations to study the impact of different factors, including the accuracy of distance measurements, the number of sensor nodes, the number of step locations, and the number of optimization rounds.

#### Metrics and ground truth

To better understand the performance of our system, we evaluate the performance of three design components (i.e., distance measurement, localization and self calibration) and their impacts on the end-to-end performance, respectively. We use the distance error (i.e., the residual error between the ground truth distance and measured distance, $e_d=|d-{\hat{d}}|$) to evaluate the distance measurement module, and use the localization error (i.e., the residual error between the ground truth position and estimated position, $e_p=\Vert p-{\hat{p}}\Vert$) to evaluate the localization and self calibration modules. For easy evaluation, we transformed the estimated relative sensor locations from the self calibrating process in a local coordinate system to align with the global coordinate system through Procrustes analysis^63^ (with a composition of translation, rotation, and reflection).

We use Kinect XBox which incorporates a human body pose recognition model^64^ to detect human bodies with its embedded depth sensor. The depth sensor by nature can provide measure of the distance and position of detected persons relative to the depth sensor, and they are recorded as ground truth. For simplicity, we mount each UWB sensor on top of a Kinect depth sensor, so that they are co-located for comparison. In addition to the ground truth from the depth sensor, we also use some fixed pre-known locations and trajectories as the ground truth for evaluation.

### Experiments with real-world testbeds

We first evaluate the performance of distance measurements with UWB monostatic radars to characterize the distance error at a single sensor node. Then, we evaluate the localization module using simultaneous distance measurements from distributed sensor nodes assuming the sensor locations are known. Finally, we evaluate the self calibrating algorithm compared to using MDS only as well as combining with MDS for initialization.

**Figure 9:**
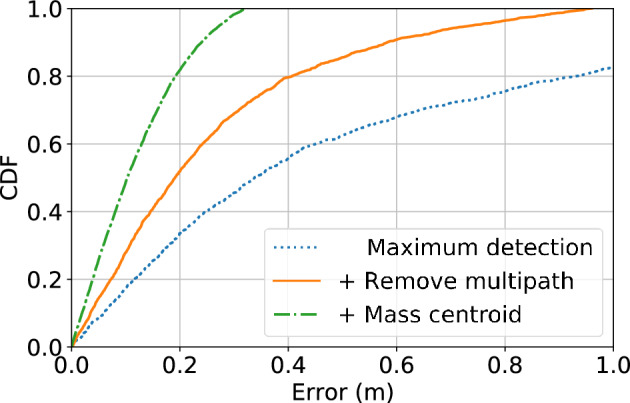
Comparison of distance measurements between methods with challenging issues addressed progressively.

**Figure 10:**
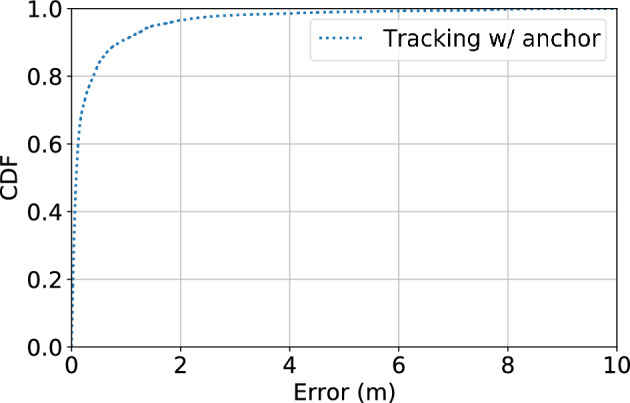
Tracking performance using distance measurements with known sensor locations.

**Figure 11:**
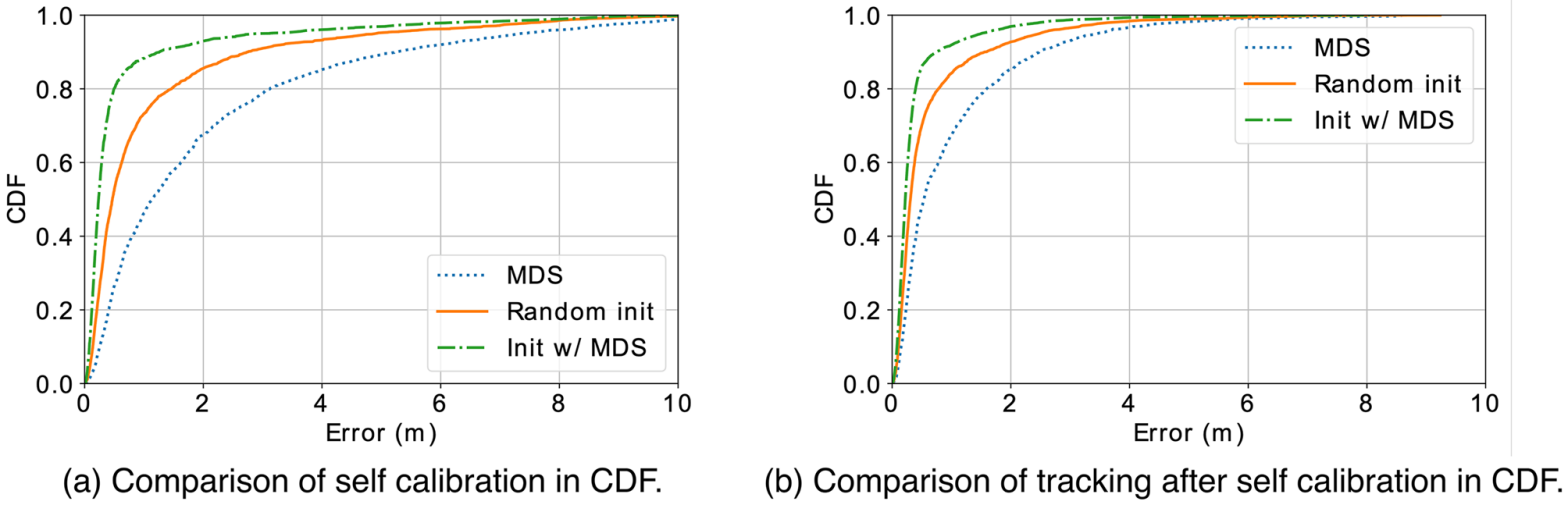
Our proposed method with random initial coordinate assignments outperforms the adapted MDS as a baseline. With MDS for initial coordinate assignment, the performance is further improved.

#### Distance measurement

Figure 9 shows CDF of distance measurement error compared to naive methods which do not address issues due to the multi-path effects and/or spread scatterer of human body. The results indicate an incremental improvement in the distance measurements by addressing challenges from the static reflection and dynamic multi-path components in the cluttered environment progressively. With all issues addressed using our method, we achieve the median error of distance measurements about 10 cm.

#### Localization

Figure 10 shows the error distribution of localization using known sensor locations, and our system can achieve a 80-percentile error of 40.1 cm, which is promising for indoor trajectory tracking and corresponding analytics. However, using known sensor locations means such performance is achieved at the cost of hours of intensive manual calibration of sensor placements, which hinders large scale deployment of such systems.

#### Self calibration

Figure 11a shows the error distribution of self calibrated sensor locations. Figure 11b shows the error distribution of tracking performance using the estimated sensor locations after self calibration. We observe that the performance of using MDS alone is not satisfying and of large variability due to its dependency on the step locations which is of large randomness. On the other hand, using MDS for initial coordinate assignment does improve the performance of self calibration, thus end-to-end tracking performance with a 80-percentile error of 40.5 cm. The proposed self calibrating method combined with the adapted MDS for initial coordinate assignments outperforms the adapted MDS as a baseline by 3.5m/1.6m in in 80-percentile error of self calibration and tracking, respectively. Observations that tracking errors are smaller than self calibration errors can be explained by that complementary information in multilateration with redundancy mitigates the impact from the error in estimated sensor locations. It is also interesting to observe that the tracking performance with noisy estimated sensor locations is comparable to the tracking performance with known sensor locations. Such an observation implies that our system can save hours of intensive manual calibration efforts at the cost of negligible degradation in tracking performance by mere 1%.

**Figure 12:**
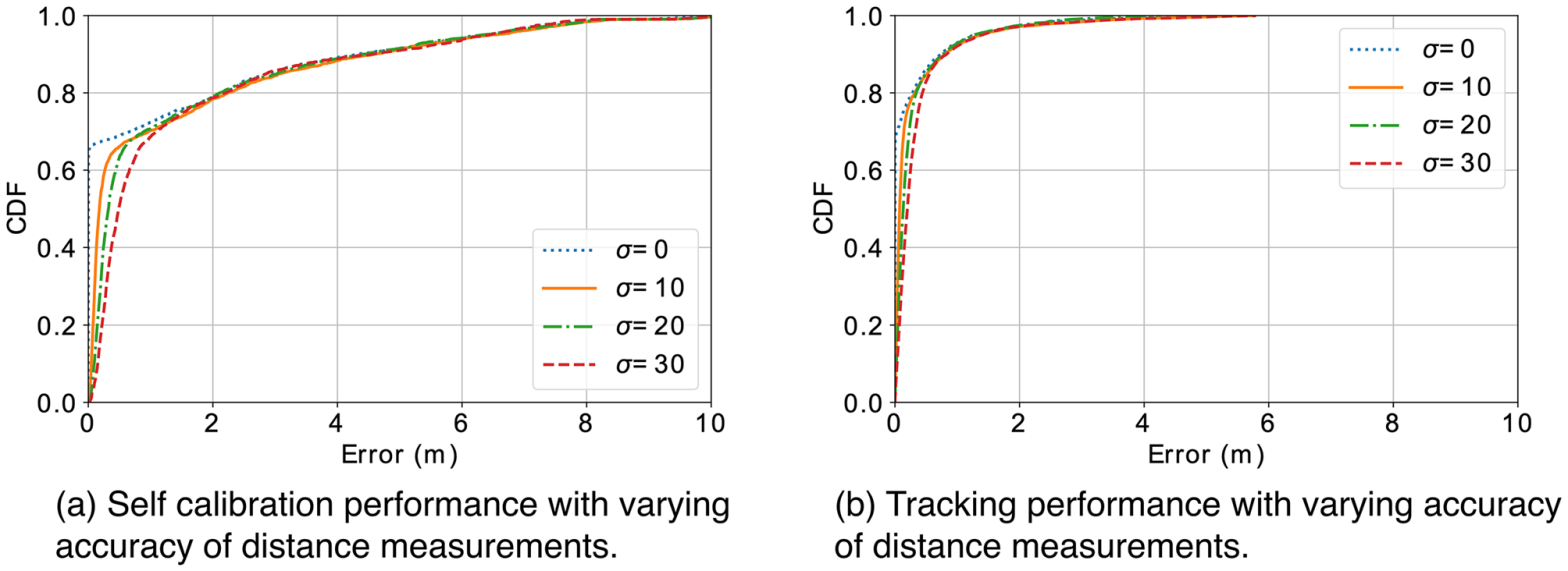
With better accuracy of distance measurements, a better performance can be achieved in both self calibration and tracking.

**Figure 13:**
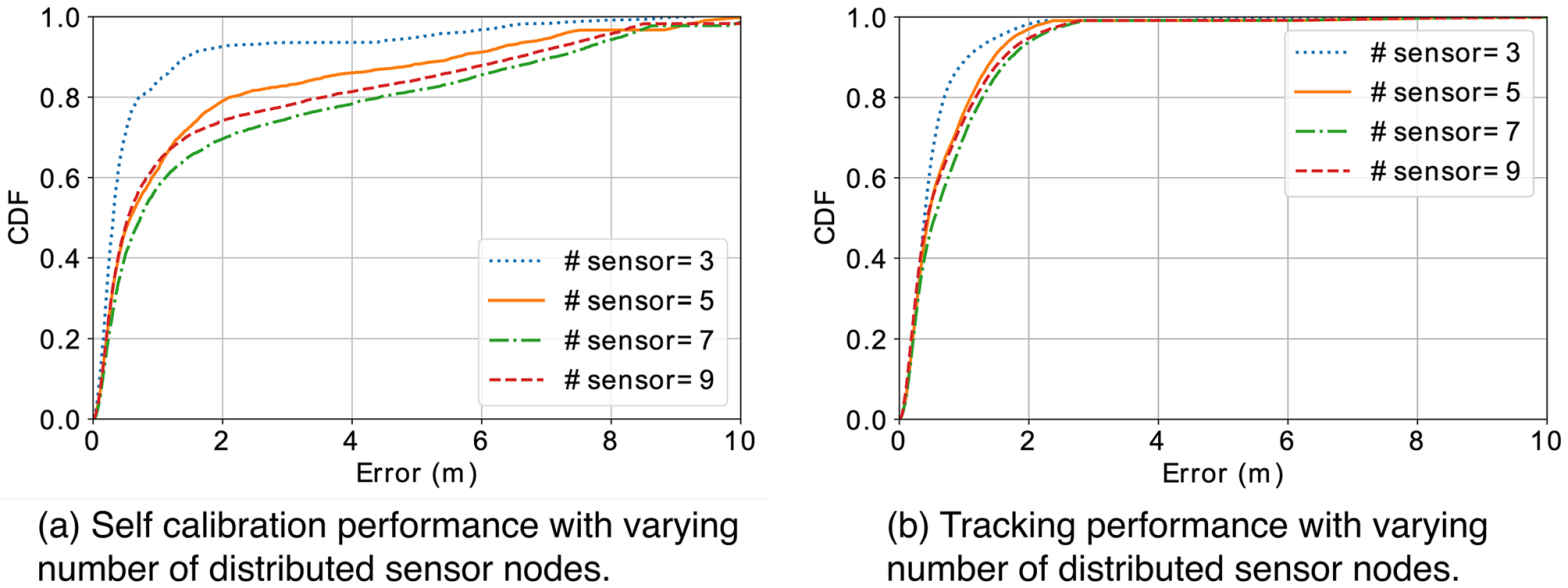
With increasing number of distributed sensor nodes, it is harder to achieve convergence of self calibration, thus worse estimate of sensor locations. However, the tracking performance is comparable among different number of sensor nodes, because the tracking error was mitigated by the redundancy of the anchors where a portion of them are well calibrated.

**Figure 14:**
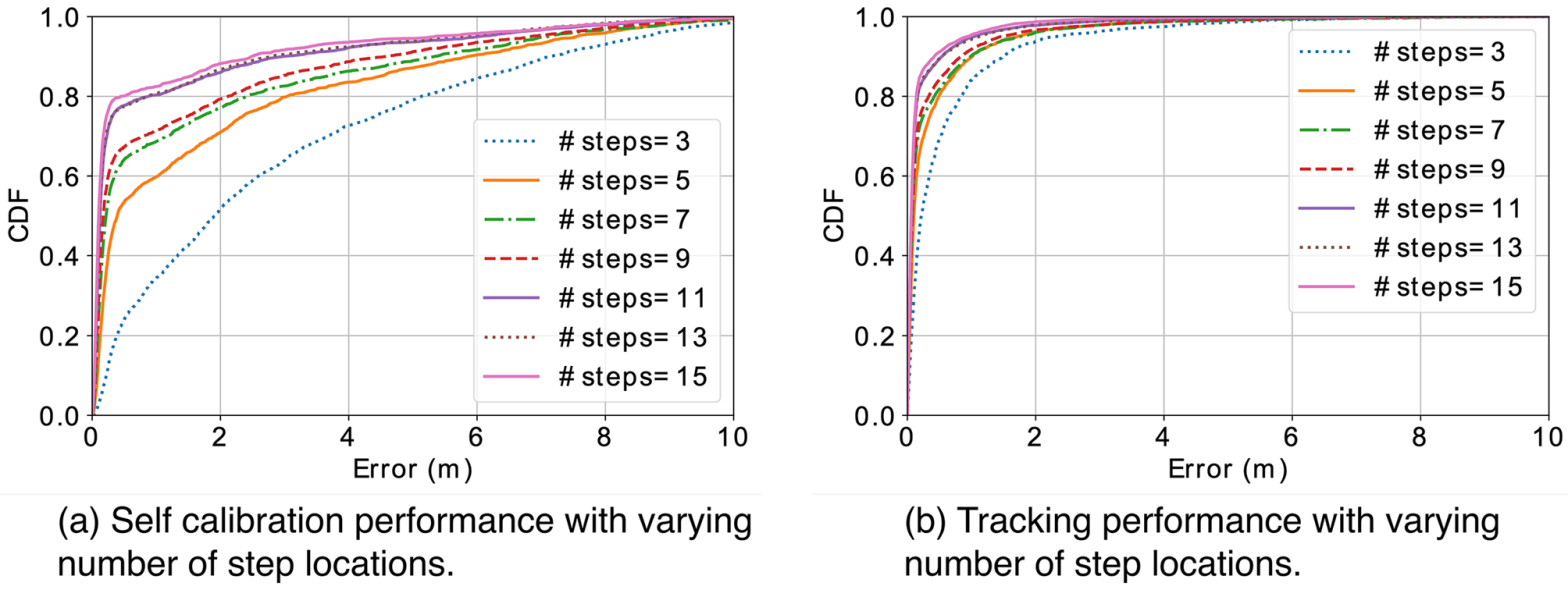
With increasing number of step locations, both self calibration and tracking are getting better performance.

**Figure 15:**
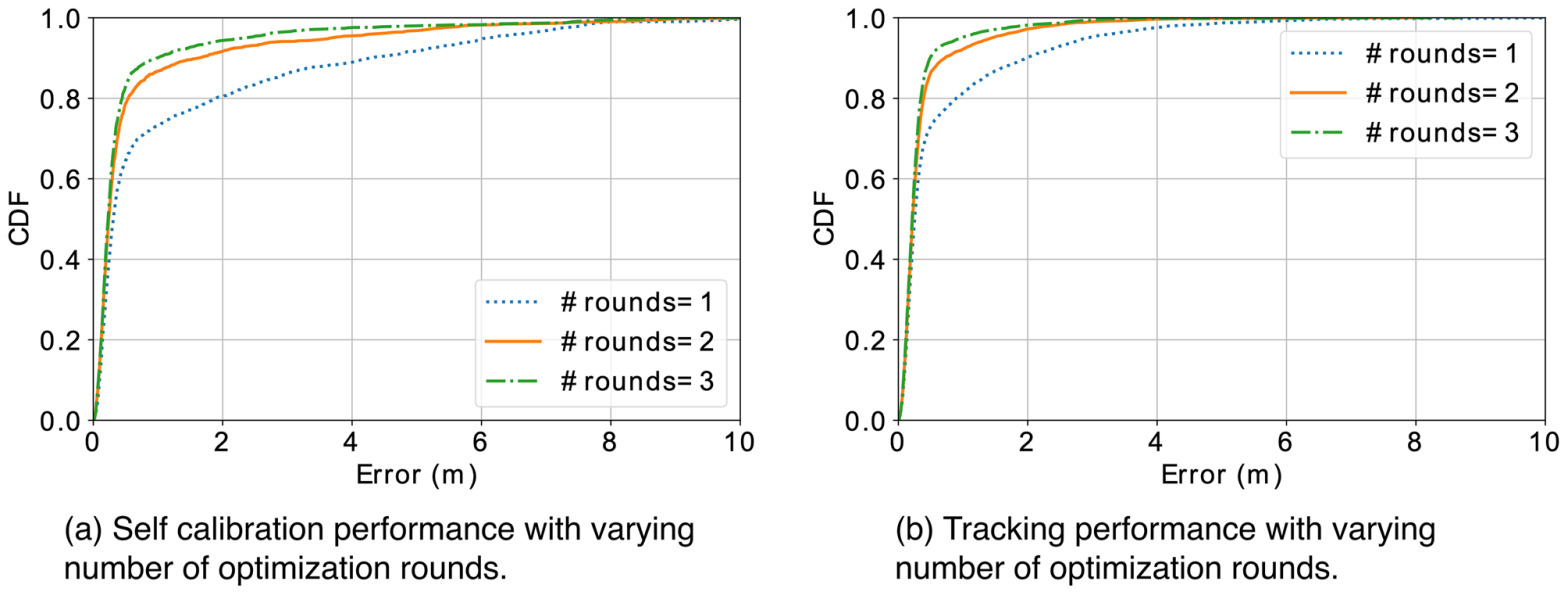
With increasing number of optimization rounds, both self calibration and tracking are getting better performance.

### Factor study with simulations

To better understand the effectiveness of our design, we conducted extensive simulations to examine the impact of different factors on the system, including the accuracy of distance measurements, the number of sensor nodes, the number of step locations, and the number of optimization rounds. To evaluate *SCALING* with simulations, we randomly generate coordinates of distributed sensors and steps, as well as noisy distance measurements between them. The default setting is configured with 5 sensor nodes, 9 step locations, 1 optimization rounds, and the Gaussian noise of 10 cm in distance measurements. When we vary one factor, the other parameters remain unchanged if not specified in the following discussion. We believe such knowledge can help form some guidelines to improve the performance and lead to more effective sensor deployment.

#### Accuracy of distance measurement

To understand how the accuracy of distance measurements will impact system, we simulate distance measurements with Gaussian noise of different standard deviations, varying from 0 to 30 cm. Figure 12a shows the performance of self calibration with different accuracy levels. Figure 12b shows the tracking performance with different accuracy levels after self calibration. Results indicate that the errors of both self calibration and tracking are related to the varying accuracy of distance measurements to some extent. However, the impact of distance measurements is limited on the end-to-end tracking accuracy (with similar 80-percentile errors around 40 cm from varying accuracy of distance measurements).

#### Number of distributed sensor nodes

In previous discussion, we conjectured that increasing the number of sensor nodes can reduce in the uncertainty in localization with multilateration. To examine the conjecture, we vary the number of distributed sensor nodes from 3 to 9. Figure 13a shows the performance of self calibration with increasing number of sensor nodes. Surprisingly, the self calibration error becomes larger with more sensor nodes. That can be explained by the fact that more sensor nodes add uncertainty in the geometrical topology, and a small portion of nodes may flex while preserving constraints in distance measurements. Figure 13b shows the tracking performance with increasing number of sensor nodes. Counter-intuitively, the tracking performance remains similar with increasing number of sensor nodes, even though their calibration performance degrades. It can be explained by the fact that multiple (redundant) anchors can compensate the noisy location of each other owe to the merit of multilateration based tracking method. This observation implies that a small number of nodes would provide sufficient accuracy, while partitioning may need to be considered for self calibration to cover a large space.

#### Number of step locations

We compare the self calibration performance with different number of step locations and the resulting tracking performance with self calibrated sensor locations. Figure 14a shows the performance of self calibration with different number of step locations. Figure 14b shows the tracking performance based on sensor locations calibrated with different number of step locations. Interestingly, we observe a consistent trend in that increasing the number of step locations improve the performance of both self calibration and tracking. Such an observation suggest using more step locations will help improve the end-to-end performance.

#### Number of optimization rounds

The optimization process for self calibration may converge at a local minimum with distorted topology due to noisy distance measurements and random step locations. Multiple rounds of optimization can help mitigate convergence at the local minimum. We observe improvements in performance of both self calibration and tracking given more rounds for self calibration in Fig. 15a and b, respectively.

### Numerical analysis of sensor placements

We conduct Monte Carlo experiments to numerically analyze the impact of sensor placements on tracking performance in the covered area. We first describe the Monte Carlo method, with details of the experimental settings, the metric of global tracking/localization performance in the area of interest, and a greedy search algorithm to approach the quasi-optimal sensor placements. Then we study the impact of sensor placements by comparing their resulting performance, and we summarize the observations and findings into guidelines for practical sensor deployment.

**Figure 16:**
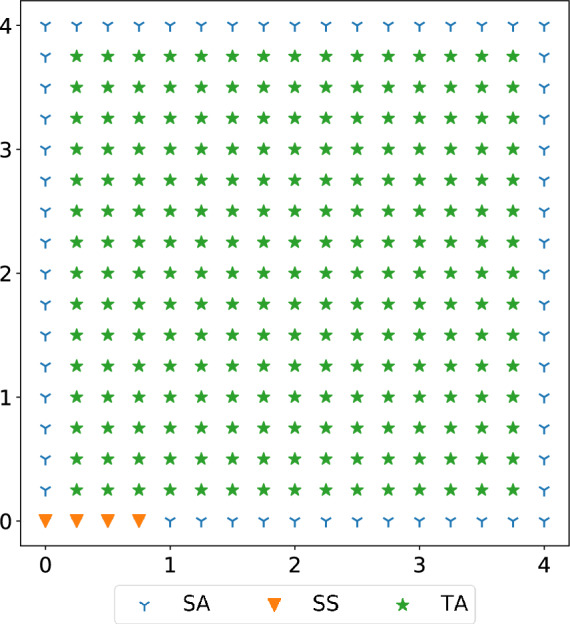
The simulated example monitoring area is of a normal room size (4 m$\times$4 m). In this setting, all possible sensor locations (SA) are along the wall/perimeter of the rectangle monitoring area. The selected sensor locations (SS) are initiated in a co-linear array at a corner indicated by the triangles in orange. The potential target locations (TA) are discrete within monitoring area indicated by green stars.

**Figure 17:**
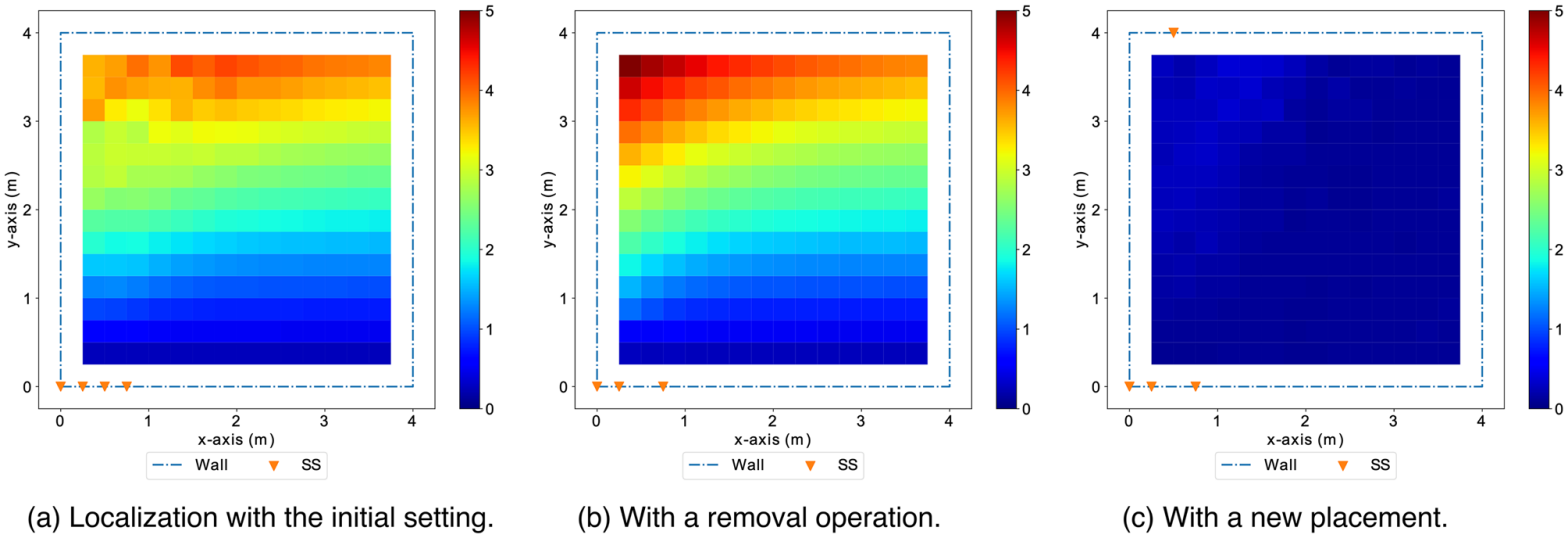
The heatmaps show the distributions of localization errors, which are dependent on the location of the target. The color of each pixel indicate the average error at the corresponding location according to the color bar (with an error range 0–5 m). In (**a**) the initial setting with 4 selected sensors (SS) in a co-linear array, the target locations in the region close to the co-linear array of sensors have smaller localization errors than distant locations. (**b**) With the least effective node removed from the initial setting, the localization accuracy degrade slightly. (**c**) With a new sensor placed at the most effective location, the average localization errors decrease to around 1 m and become more uniformly distributed across all locations.

**Figure 18:**
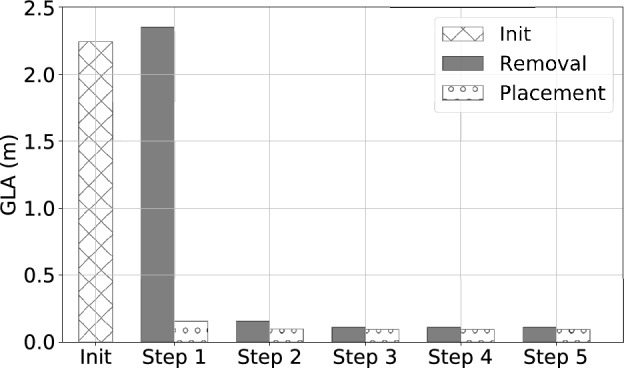
The global localization accuracy (*GLA*) jumps largely from $\sim 2$ m to $\sim 0.2$ m at the first placement, and then converges at a small *GLA* ($\sim 0.1$ m) along greedy search steps with alternating removal and placement operations.

**Figure 19:**
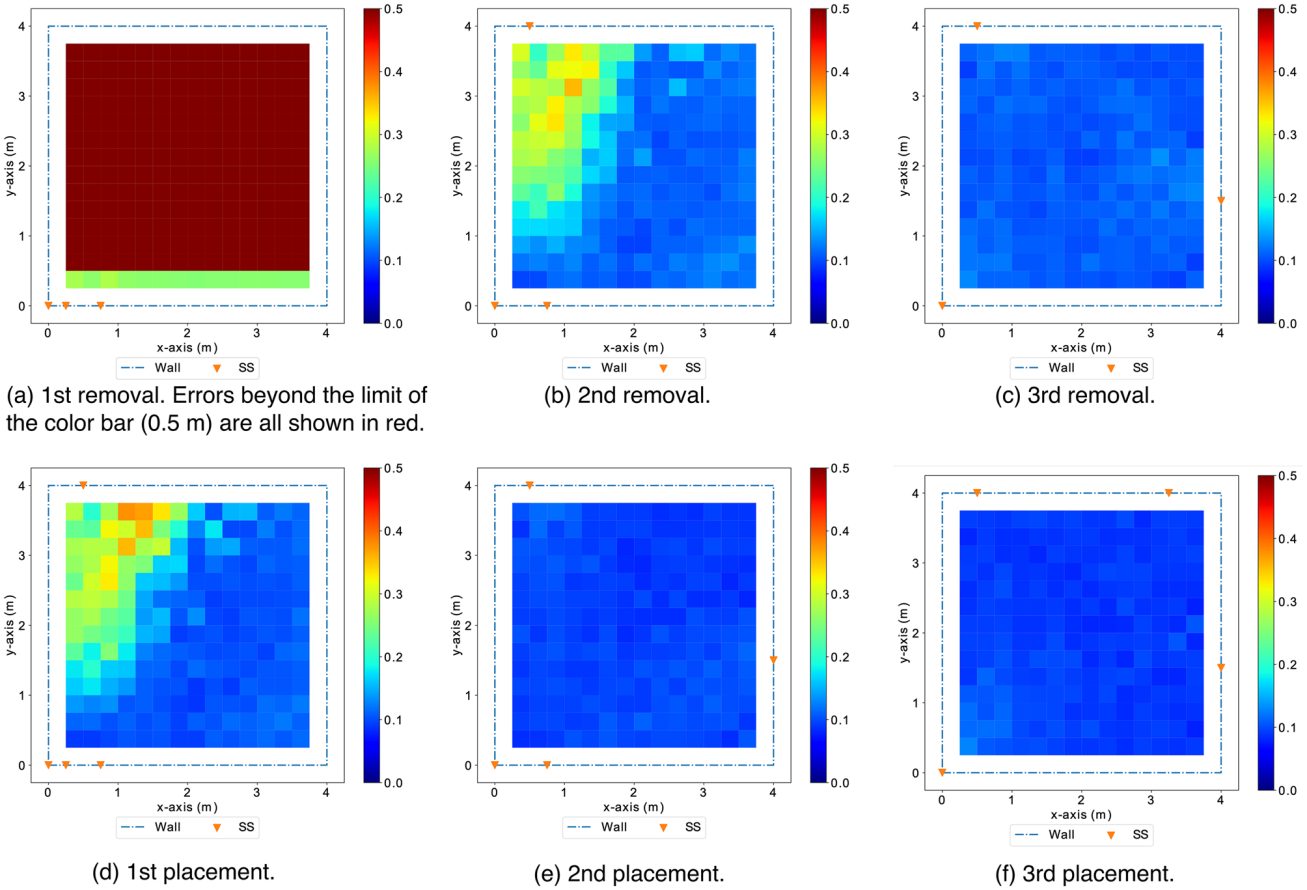
The heatmaps show distributions of localization errors according to the color bar with an error range 0-0.5 m (with any error values beyond 0.5 m all in red) in different configurations of sensor placements. The least effective sensor nodes identified in removal steps (**a**–**c**) are those contribute the least space diversity to the previous configurations. The most effective sensor nodes to be placed in alternating steps (**d**–**f**) are distant from the existing sensor nodes, adding significant space diversity to the existing configurations.

#### Monte Carlo method

In Monte Carlo experiments, we first define the covered area in 2D plane, usually a room-sized rectangle confined by length *l* and *w*. Figure 16 shows an example setting. We envision that sensors will be mounted on walls in real homes where the human subjects move in between. Therefore, we set the sensor nodes on the perimeter of the rectangle, and the target to be localized within the rectangle. To balance the computational complexity and the resolution of evaluation results, we follow the experimental settings in the related work^8^ to set the minimum antenna separation as 0.25 m in either x-axis or y-axis along the perimeter of the generated rectangle. Similarly, we divide the monitoring area into a grid of discrete cells of the size 0.25 m by 0.25 m to set potential target locations. For each configuration of sensor placements, we evaluate the localization accuracy in all potential target locations; and for each target locations, we repeat *Q* times the distance measurements with Gaussian noise for multilateration localization. Instead of using the localization accuracy in a single location—local localization accuracy (*LLA*), we use a new metric—global localization accuracy (*GLA*), that is the average of localization error in all the potential target locations within the area of interest, expressed as:

$$\begin{aligned} GLA=\frac{1}{A}\sum _{i=1}^A {LLA_{i}} =\frac{1}{A}\sum _{i=1}^A (\frac{1}{Q}\sum _{j=1}^{Q}\left( \Vert {\dot{p}}_{i}-{\hat{p}}_{i,j}\Vert \right) ), \end{aligned}$$

where *A* denotes the total number of potential target locations in the area of interest, *i* denotes the index of target location, *Q* denotes the number of times we repeat the distance measurements, and *j* denotes the index of repeated experiments for a certain target location index *i*.

To numerically analyze the impact of sensor placements and develop guidelines for achieving a quasi-optimal configuration in practical sensor deployment, we compare the tracking/localization performance between various configurations of sensor placements according to the metric of *GLA*. In Monte Carlo experiments, we use the Gaussian noise of 10 cm in distance measurements, close to the parameters of realistic distance measurements. Given *M* sensor nodes at *L* potential placements and *L* is much larger than *M*, the computational complexity for finding the optimal configuration is high with the combinatorial search ($${\mathscr {O}}\left( {\begin{array}{c}M\\ L\end{array}}\right)$$). Instead, we leverage a greedy algorithm to approach the optimal sensor placements progressively from an initial configuration $S_0$ with a reduced computational complexity (${\mathscr {O}}(M\cdot L$)). The greedy algorithm is described in below steps:

1. We calculate the tracking performance $GLA_0$ of the initial configuration $S_0$ in a predefined area of interest.
2. By repeatedly removing one out of *M* sensor nodes in the previous configuration ($S_0$), we search for the node of which the removal leads to the least decrease in *GLA* compared to $GLA_0$, and this sensor node corresponds to the least effective one among *M* sensor nodes (illustrated in Fig. 17b).
3. After removing the least effective node from $S_0$, we alternatively add one sensor node to a new placements. We repeatedly vary the placements of the new node to find the one with the most increase in *GLA* compared to $GLA_0$, and that mean we find the most effective placement for the new node (illustrated in Fig. 17c). With this new sensor node, we denote the current configuration of sensor placements as $S_1$ and its tracking performance as $GLA_1$.
4. We progressively update the sensor placements by repeatedly alternating removal and placement operations described in steps 2 and 3, until the variation in *GLA* between consecutive configurations is less than a small threshold.

Figure 16 shows the initial setting, where we set the monitoring area as a 4 m by 4 m rectangle and we initially place 4 sensor nodes at the corner of the monitoring area to emulate the case where the sensor nodes were configured in a co-linear array^8^. For statistical consistency, we empirically repeat 100 times of distance measurements for multilateration localization at each locations in the covered area.

**Figure 20:**
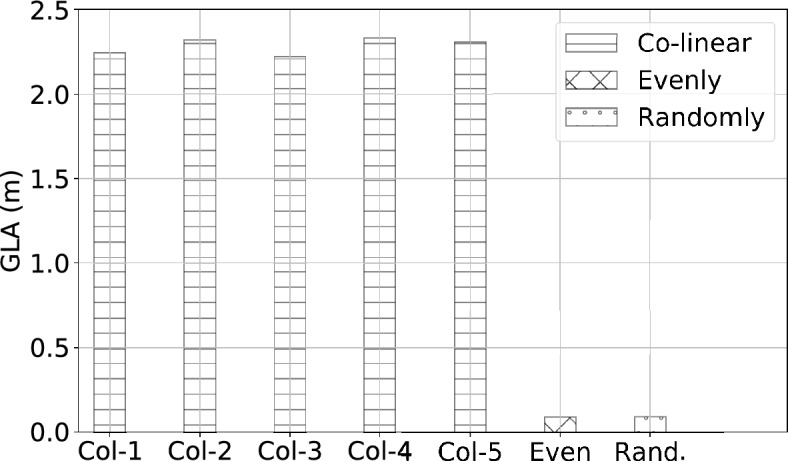
The bar plot shows *GLA* in different configurations of sensor placements. The co-linear arrays with varying separation spaces (1–5 separation units) between sensor nodes all have similar *GLA* around 2.2 m. The configuration with sensors evenly distributed at 4 corners has a satisfactory accuracy ($\sim 0.1 m$), and the configuration with sensors randomly spread at 4 walls has a similar GLA to the configuration with sensors evenly distributed.

**Figure 21:**
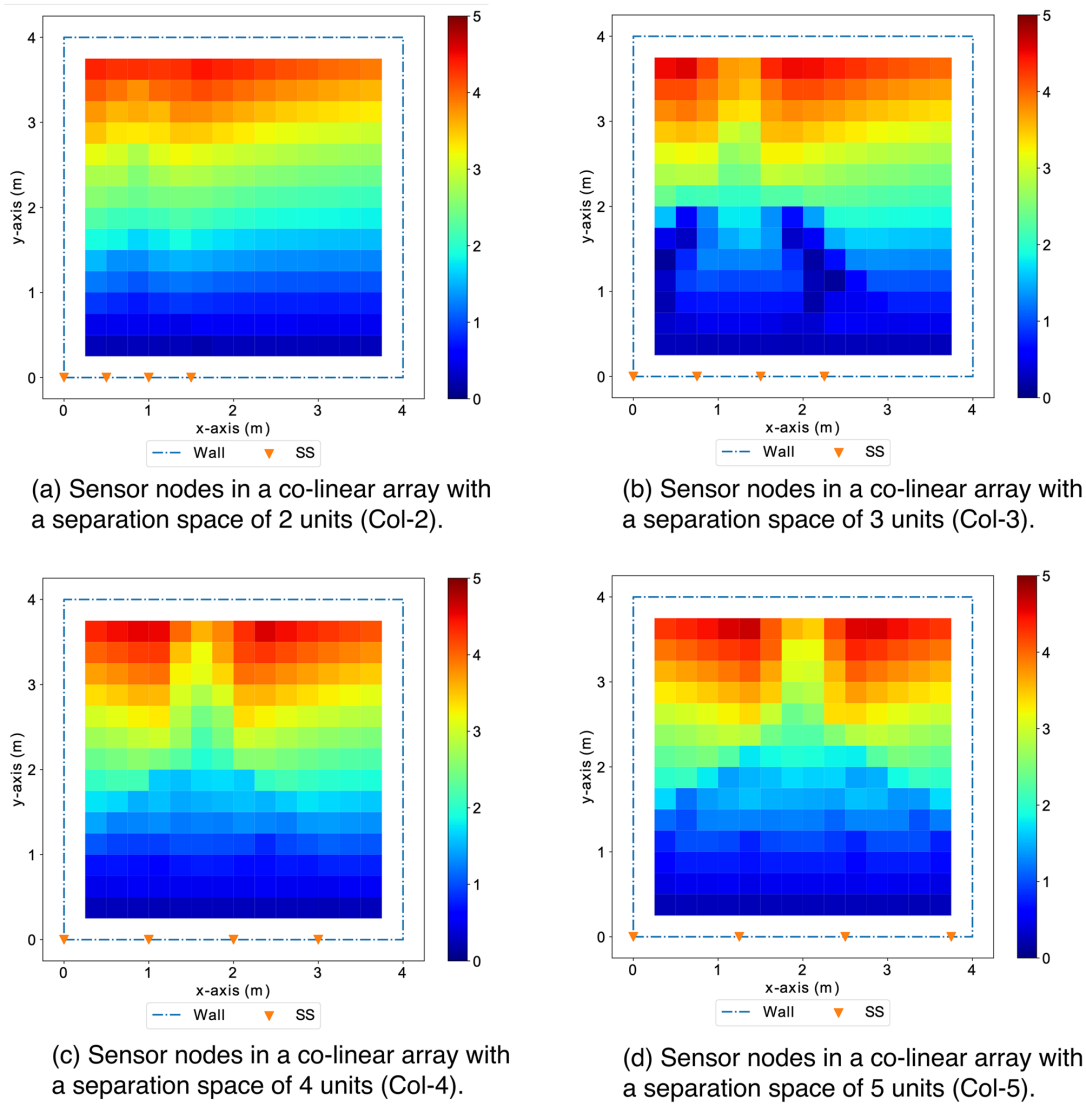
Co-linear arrays with different separation space between sensor nodes show similar tracking/localization performance (with *GLA* of $\sim 2.2$ m errors). The distribution of localization errors across the monitoring space presents a beamforming-like pattern symmetric to the co-linear array and the region near the co-linear array has better accuracy than farther region.

**Figure 22:**
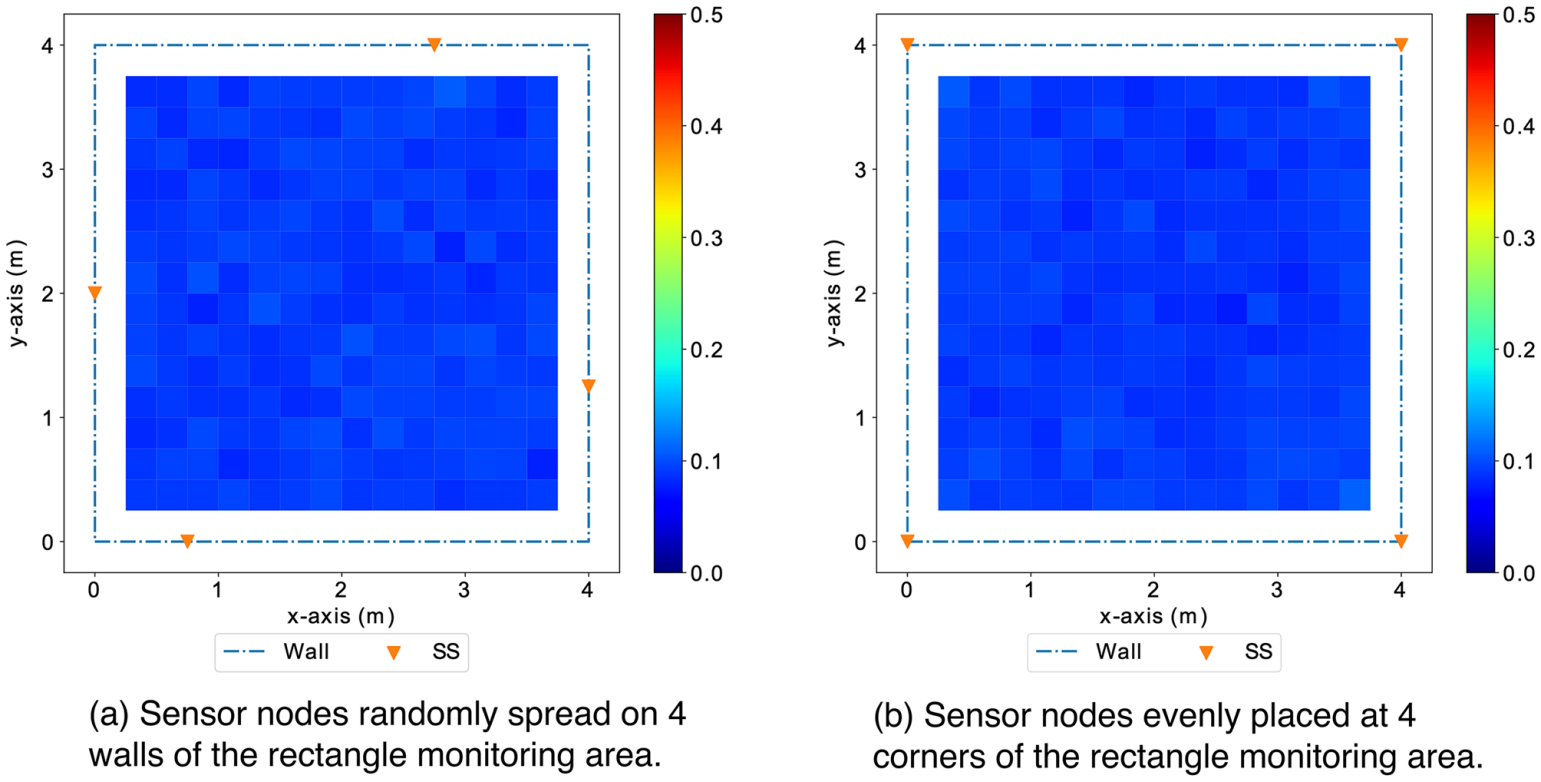
The configurations (a) with 4 sensor randomly spread on walls surrounding the monitoring area has a comparable performance to the configuration with sensors evenly distributed at 4 corners of the rectangle monitoring area.

#### Impact of sensor placements

Figure 17 shows the distribution of localization errors across the monitoring area with the initial setting, a removal of the least effect node, and a placement of the most effective node in respective heatmaps. Figure 18 shows the trend of *GLA* along the greedy search steps with alternating removal and placement operations. Figure 19 shows heatmaps, which indicate the distributions of localization errors with different sensor placements along the greedy search process. We observe that the sensor nodes that are less effective are those contribute less space diversity (e.g., in the middle of a co-linear array). Similarly, the sensor placements that contribute more to the tracking/localization performance are those add the most to the space diversity (e.g., distant from the existing sensor placements).

In addition to using greedy search to approach the quasi-optimal, we also study the impact of varying separation spaces between sensor nodes in co-linear arrays. Finally, we study the performance of the configurations with sensor evenly distributed as well as randomly distributed. Figure 20 shows the co-linear arrays with varying separation spaces between sensor nodes are of similar *GLA* ($\sim 2.2$ m error), which are unsatisfactory for practical applications and much worse compared to the accuracy ($\sim 0.1$ m error) achieved with the configurations with sensor evenly or randomly distributed surrounding the monitoring area. This observation motivates and supports the choice of the distributed scheme for localization. Figure 21 shows the distribution of localization errors from the configurations in co-linear arrays is inconsistent across the monitoring area, and the varying separation spaces have no significant impact on the overall performance. In contrast, the configurations with sensor nodes spread evenly or randomly surrounding the monitoring area achieve satisfactory performance with consistent distribution shown in Figure 22.

**Remarks on sensor placements** The configuration of sensor placements in the co-linear array, widely adopted in existing work, may be convenient for installation and calibration, but it leads to unsatisfactory performance with inconsistent distribution of localization accuracy dependent on the target location. In general, the configurations with sensor nodes distributed sparsely surrounding the monitoring area can achieve satisfactory performance (with *GLA* around 0.1 m). The above observations motivate the distributed scheme adopted in this study, and it serves as guidelines for sensor deployment. While it may be impractical to place sensor nodes evenly in real life scenarios, a random distribution of sensor placements surrounding the monitoring area with large separation usually provides a sufficient performance.

## Discussion

In this section, we discuss the limitations and opportunities to guide future work.

**Scalability.** For clarification, the term “large scale deployment” in this paper specifically refers to scaling deployments to a good number (e.g., tens or hundreds) of homes of diverse layouts. Covering one site with large indoor spaces (e.g., a transportation hub) is a different issue: while self calibration with a small number (e.g., 3–5) of sensor nodes can be reasonably accurate, more nodes do not necessarily lead to better performance due to increasing uncertainty of sensor placements. In the future, we will explore dynamically partitioning a large area and optimizing the self calibration in each partition (e.g., with only three closest nodes). To adapt *SCALING* to co-habiting scenarios, we can follow the previous work to achieve multi-user tracking using successive interference cancellation^9^.

**Compatibility and extensibility with various RF devices** The choice of the COTS UWB used in this paper is a trade-off between several factors, including its range resolution for distance measurements, orthogonality for simultaneous sensing of distributed nodes, and low price. With this hardware choice, *SCALING* demonstrates its generazability with RF sensors of minimum requirements. It is noted that such a trade-off is achieved within the scope of COTS RF devices. We can further optimize such a trade-off with our customized RF design. Ingoring practical concerns about the cost, *SCALING* is compatible with other COTS RF techniques, and can be easily extended when additional information becomes available. Three possible cases are: (1) a better accuracy can be achieved using RF techniques of finer range resolution, e.g., mmWave. (2) When using RF sensors configured with phased array antennas instead of a single transceiver, additional AoA measurements can be integrated to further reduce the ambiguity in self calibration and localization. (3) When the cross-communication between nodes is available, we can apply the classical MDS with a complete distance matrix instead of the approximate one for more reliable initial coordinate assignment.

**Daily indoor trajectory analysis** We plan to scale data collection to tens or hundreds of real homes with easy self-installation enabled by *SCALING* so that we can focus more on health analytics with long-term daily indoor trajectories. We believe daily indoor trajectories would carry more information than just the occupancy state of inhabitants in a certain space. For example, daily trajectories can be used for profiling the ambulation ability of inhabitants, and changes in patterns such as decreasing activities in the kitchen might indicate declines in cognitive and physical abilities, early indicators for people at risks of diseases like Alzheimer’s.

## Conclusion

We propose *SCALING*, a plug-and-play device-free indoor trajectory monitoring system that a layman user can easily set up by one-minute walking. It uses a self calibrating algorithm to save hours of intensive manual efforts and technical expertise at the cost of negligible degradation in end-to-end performance. We believe *SCALING* opens door for large scale deployment of in-home monitoring system, manageable by relatively small size research teams, thus potentially benefiting large populations beyond a handful of homes.

## Data Availability

The data that support the findings of this study may be made available from the corresponding authors after further processing and applicable review on intellectual property upon requests for academic purposes.
